# The red box: a selective G-quadruplex binder that downregulates *c-MYC* expression in human cells

**DOI:** 10.1039/d5cb00329f

**Published:** 2026-08-18

**Authors:** Paula Novo, Iván Montes de Oca, Claudia García-Delgado, Agustín Sánchez-Temprano, Elena Pazos, Carlos Peinador

**Affiliations:** a CICA – Centro Interdisciplinar de Química e Bioloxía and Departamento de Química, Facultade de Ciencias, Universidade da Coruña A Coruña 15071 Spain; b Oportunius, Axencia Galega de Innovación 15702 Santiago de Compostela Spain elena.pazos@udc.es carlos.peinador@udc.es

## Abstract

G-quadruplex (G4) structures within oncogene promoters represent promising therapeutic targets, particularly in the context of transcriptional regulation. Here, we report that the red box, a fully organic macrocyclic cationic receptor, exhibits high affinity and selectivity for G4 structures. Biophysical analyses reveal that the red box binds the *c-MYC* G4 with nanomolar affinity. Notably, the red box displays greater selectivity for *c-MYC* over duplex DNA compared to the well-characterized porphyrin TMPyP4, and its macrocyclic architecture is essential for this activity, as its acyclic analogue displays negligible G4 stabilization. In cellular assays, treatment with non-cytotoxic concentrations of the red box selectively downregulated *c-MYC* expression in HEK293T cells, with minimal effects on *c-KIT* and *hTERT*. Unlike previously reported G4-selective ligands based on Pd(ii)/Pt(ii) metallacycles, the red box achieves nanomolar affinity and selectivity through a fully metal-free, hydrazone-based scaffold, addressing key limitations related to cost, biodegradability, and long-term biocompatibility. Furthermore, *c-MYC* downregulation was consistently observed across multiple cell lines, including tumour-derived cellular models, underscoring the translational relevance of this new class of organic G4 binders.

## Abstract 2

Red box, a fully organic macrocycle, binds the *c-MYC* G-quadruplex with nanomolar affinity and high selectivity over duplex DNA, and selectively downregulates *c-MYC* expression in cells.

## Graphical abstract

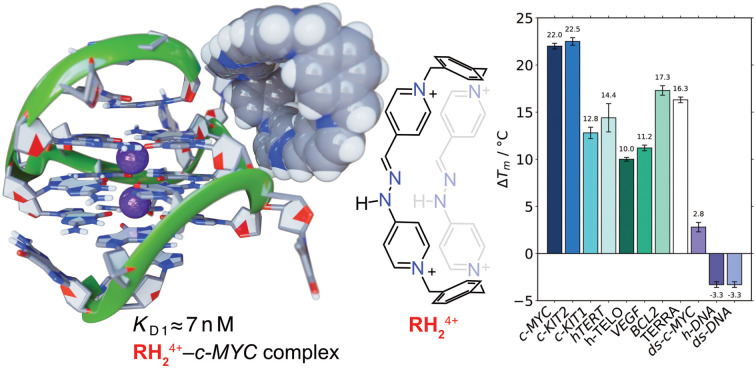

## Introduction

G-quadruplex DNA (G4) is a well-characterized non-canonical secondary structure formed by guanine-rich nucleic acid sequences, and was first identified nearly four decades ago.^1,2^ These structures commonly arise in genomic regions with high guanine content, such as telomeres and promoter regions of certain genes, as well as in guanine-rich RNA segments. Under physiological conditions, G4-forming sequences fold into stacked planar arrangements of four guanine bases, known as G-tetrads or G-quartets, which are stabilized by Hoogsteen hydrogen bonds and coordinated monovalent cations, typically K^+^ or Na^+^.^3^ These structures display substantial topological diversity, and unimolecular G4s generally adopt parallel, antiparallel, or hybrid conformations.^4^ Biologically, G4 structures are implicated in key regulatory processes such as telomere length maintenance and regulation of gene expression. Consequently, dysregulation of these processes is associated with various pathologies, including cancer.^5,6^ A notable example is the proto-oncogene *c-MYC*, which contains a G4-forming sequence in its promoter region^7^ and is overexpressed in approximately 80% of solid tumours. This overexpression is closely linked to abnormal cell growth, proliferation, and impaired apoptotic regulation.^8^ Similarly, the proto-oncogene *c-KIT*, which also contains a G4-forming sequence in its promoter region, encodes a tyrosine kinase receptor involved in the regulation of cell growth and proliferation and is frequently overexpressed or mutated in malignant cancers, such as gastrointestinal stromal tumours.^9^

In this context, G4 motifs within genomic regions associated with carcinogenic processes have become attractive therapeutic targets. Over the past two decades, efforts to design and develop molecules that selectively bind and stabilize G4 structures have expanded rapidly, driven by the goal of exploiting their anticancer potential.^6,10–12^ Among the diverse classes of G4-targeting ligands explored for anticancer treatments,^13–17^ macrocyclic compounds have attracted particular interest due to their structural versatility and capacity for selective molecular recognition. Traditionally employed as synthetic receptors in host–guest chemistry, many macrocycles are well-suited for interacting with G4 DNA. Notable examples include the natural macrocycle telomestatin,^18^ the cationic porphyrin TMPyP4,^19^ or the macrocycle BOQ-1,^20^ among others. Outstandingly, these compounds bind G4 DNA with high affinity while exhibiting varying degrees of selectivity over duplex DNA. Their recognition properties arise from a combination of π–π stacking, hydrogen bonding, and electrostatic interactions. Building on the success of covalent macrocycles, attention shifted towards metal-coordinated macrocyclic systems, particularly due to their synthetic simplicity and structural complementarity with G4 structures. Coordination-driven self-assembly enabled the efficient construction of architectures whose size and geometry can be tailored to match G4 DNA topologies. Among these, Pd(ii) and Pt(ii)-based self-assembled metallacycles emerged as potent G4 ligands, showing high affinity and selectivity.^21–25^ However, their broader biomedical application faces many potential drawbacks. Palladium and platinum are costly, scarce, and non-biodegradable, and may accumulate in tissues overtime, raising concerns regarding long-term safety, environmental impact and economic viability.

To overcome some of the limitations associated with the Pt(ii)-based metallacycles previously developed in our group,^24–28^ which resemble the family of pyridinium-based polycationic cyclophanes (commonly referred to as ExBoxes due to their box-like geometry) originally developed by Stoddart and co-workers,^29^ we have lately explored the use of hydrazone bonds as a synthetically efficient alternative to metal coordination. This strategy has enabled the construction of fully organic analogues, such as the so-called white box and red box.^30–32^ Remarkably, the red box analogue, which incorporates hydrazone rather than acylhydrazone bonds, showed enhanced aqueous stability, likely due to charge delocalization from the amine lone pair across the pyridinium rings. Given its ability to form inclusion complexes with electron-rich aromatic substrates in water and its structural similarity to the aforementioned Pt(ii) metallacycles, we hypothesized that this polycationic cyclophane could act as a G4 DNA binder.

In this context, the present work shows that the red box is the first fully organic, hydrazone-based ExBox analogue capable of acting as a G4-binding agent. We demonstrate that it selectively interacts with a panel of biologically relevant G4-forming oligonucleotides, displaying a marked preference for the *c-MYC* and *c-KIT2* G4s. Importantly, the red box exhibits significantly strong selectivity for G4 structures over duplex DNA. Moreover, biological assays have shown that this cyclophane significantly downregulates the expression of *c-MYC* in multiple cellular models. These findings establish the red box as a privileged G4 ligand, metal-based or metal-free, which combines nanomolar *c-MYC* affinity, selectivity over duplex DNA exceeding that of TMPyP4, and reproducible transcriptional downregulation across four independent human cell lines. This functional breadth, rarely demonstrated even among well-established G4 binders, is achieved here through a fully organic, hydrazone-based scaffold, expanding the design space for G4-targeted gene regulation beyond metal-coordination chemistry.

## Results and discussion

### Structural features of the red box for G4 recognition

**Fig. 1:**
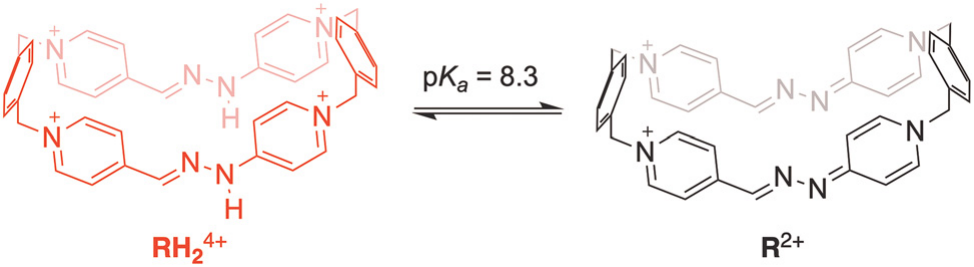
Depiction of the red box macrocycle RH_2_^4+^ and its conjugate base R^2+^.

Ligand design plays a pivotal role in optimizing the binding properties of a selective, and ideally specific, G4 binder. In this context, the red box (RH_2_^4+^) exhibits several key structural features that align with those of the Pt(ii)-metallacycles developed by our group, as well as other macrocycles reported in the literature. First, RH_2_^4+^ features an electron-deficient aromatic macrocyclic scaffold (Fig. 1), which can promote π–π stacking interactions with G-quartets through end-stacking. Second, the rigidity and dimensions of these scaffolds enhance shape complementarity with the G-quartet surface, as shown for Pt(ii)-metallacycles,^23,24^ while hindering intercalation into duplex DNA. Third, the presence of positive charges within the macrocycle facilitates electrostatic interactions with the negatively charged phosphate backbone of G4 structures. Together, these features make RH_2_^4+^ a structurally well-suited candidate for selective and efficient G4 binding.

To evaluate the G4-binding properties of RH_2_^4+^ in a biologically relevant context, we selected a panel of G4-forming nucleic acid sequences and duplex DNA controls (Table S1). The G4 panel included promoter G4s from oncogenes and cancer-related genes (*c-MYC*,^33^*c-KIT1*,^9^*c-KIT2*,^34^*BCL2*,^35^*VEGF*,^35^ and *hTERT*^36,37^), the human telomeric sequence h-TELO,^38^ and the RNA G4 TERRA.^23^ Binding selectivity was assessed using several duplex DNA controls, including ds-*c-MYC*, a 20-mer hairpin duplex DNA (h-DNA), and a 20 bp duplex DNA sequence lacking loop structures.^39^

### The red box preferentially binds *c-MYC* and *c-KIT2* G4s with high affinity over other G4 and duplex sequences

**Fig. 2:**
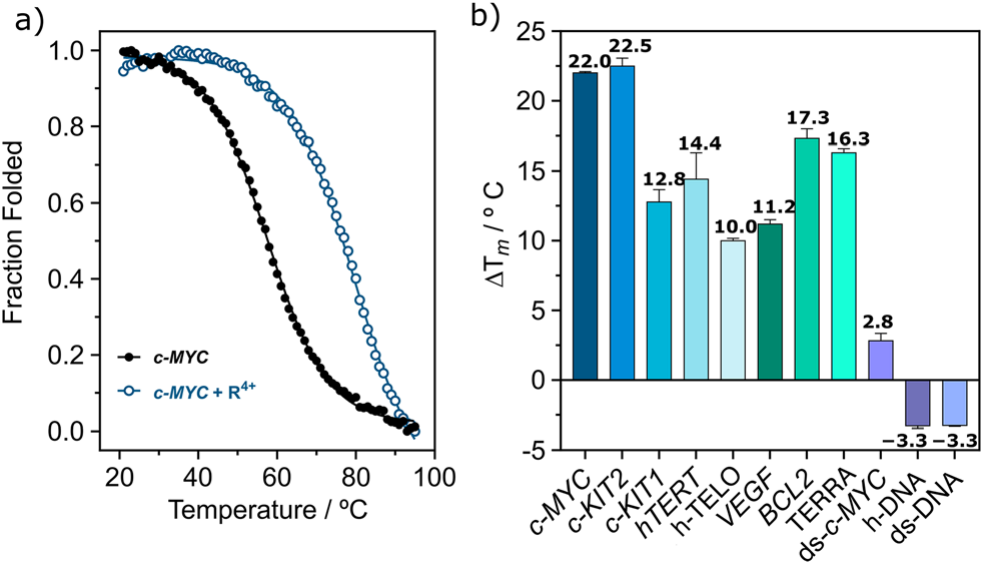
(a) RH_2_^4+^ CD melting curves at 263 nm of a 10 µM *c-MYC* solution in 10 mM lithium cacodylate, 1 mM KCl, 99 mM LiCl, pH 6.3 (LCB3), both in the absence (•) and in the presence of RH_2_^4+^ (3 eq., 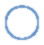). (b) Representation of the RH_2_^4+^-induced stabilization in terms of Δ*T*_m_ (values above the bars) for the selected G4 and duplex DNA sequences.

To explore the interaction between RH_2_^4+^ and the G4-forming oligonucleotides, circular dichroism (CD) melting assays were performed to evaluate G4 stabilization in the presence of the ligand. Given that the red box displays pH-responsive behaviour (Fig. 1) and approximately 15% of the macrocycle is present as the deprotonated species (R^2+^) at physiological pH, initial experiments were conducted with the *c-MYC* G4 sequence at pH 6.3, where RH_2_^4+^ is the predominant species. As the ligand does not display any intrinsic CD signal under these conditions (Fig. S1), structural changes in the G4 can be monitored directly. Since lithium cacodylate buffer is commonly used in G4 melting studies and phosphate buffers exhibit significant temperature-dependent pH variations, CD spectra of the *c-MYC* G4 were first recorded in both phosphate (10 mM potassium phosphate, 88 mM KCl, pH 6.3) and cacodylate buffers (10 mM lithium cacodylate, 1 mM KCl, 99 mM LiCl, pH 6.3).^40–43^ Identical spectra were obtained in both buffers, consistent with the characteristic signature of a parallel *c-MYC* G4 (Fig. S2).^44^ CD melting experiments were then carried out for *c-MYC* in cacodylate buffer at pH 6.3, both in the absence and in the presence of three equivalents of RH_2_^4+^ (Fig. 2a). Addition of the ligand increased the melting temperature of the *c-MYC* G4 by 22.0 °C (Fig. 2b), demonstrating strong stabilization of the G4 structure.

To evaluate the stabilization effect of RH_2_^4+^ across the other G4-forming sequences, a CD melting screening was conducted at pH 6.3 using *c-KIT1*, *c-KIT2*, *hTERT* and h-TELO, *BCL2*, *VEGF*, and TERRA (Fig. S3–S9). As described for *c-MYC*, melting curves were recorded in the absence and presence of three equivalents of RH_2_^4+^. As summarized in Fig. 2b, RH_2_^4+^ stabilized all G4 structures examined, although to different extents. The largest thermal stabilization was observed for *c-KIT2* (Δ*T*_m_ = 22.5 °C), comparable to that of *c-MYC* (Δ*T*_m_ = 22.0 °C), whereas lower stabilization was observed for h-TELO and *VEGF*. Overall, these results indicate that RH_2_^4+^ can recognize different G4 topologies, including parallel, hybrid, and RNA G4 structures, but displays a preference for selected G4 targets, particularly *c-MYC* and *c-KIT2*.

Accordingly, to assess the selectivity of RH_2_^4+^ for G4 structures, CD melting experiments were also performed using a selection of duplex DNA models at pH 6.3 (Fig. S10–S12). As represented in Fig. 2b, the impact of RH_2_^4+^ on the stabilization of ds-*c-MYC* was almost one order of magnitude lower than that measured for its corresponding G4 counterpart. In contrast, a slight destabilization was observed for both h-DNA and the ds-DNA upon addition of RH_2_^4+^. Taken together, these results indicate that RH_2_^4+^ stabilizes G4 structures much more effectively than duplex DNA.

**Fig. 3:**
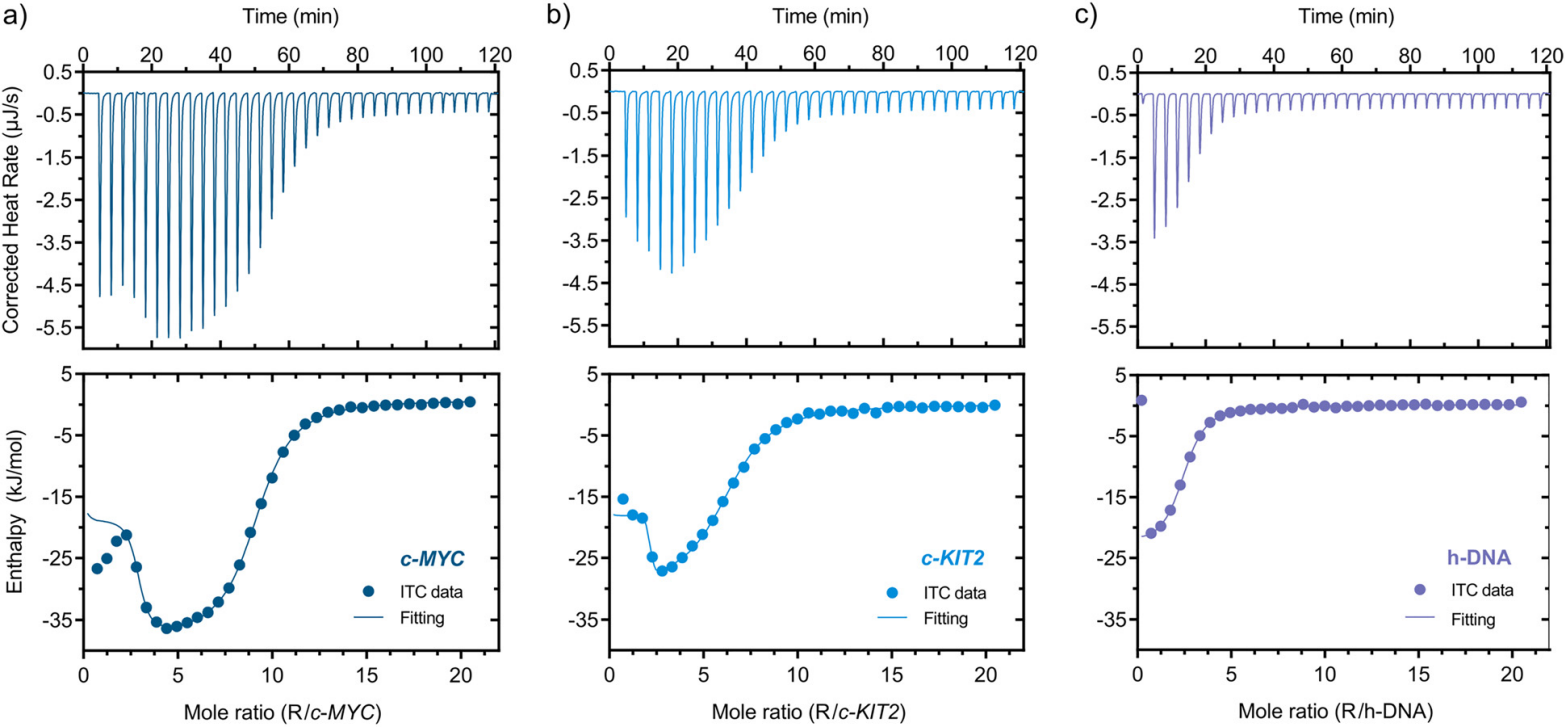
(a) ITC profile and data fitting (below) of a 10 µM *c-MYC* solution in 10 mM lithium cacodylate, 1 mM KCl, 99 mM LiCl, pH 7.2 (LCB5), upon addition of successive aliquots of a 700 µM **R** solution in the same buffer. (b) ITC profile and data fitting (below) of a 10 µM *c-KIT2* solution in 10 mM lithium cacodylate, 10 mM KCl, 90 mM LiCl, pH 7.2 (LCB6), upon addition of successive aliquots of a 700 µM R solution in the same buffer. (c) ITC profile and data fitting (below) of a 10 µM h-DNA solution in LCB6, upon addition of successive aliquots of a 700 µM R solution in the same buffer.

To obtain quantitative insight into the interaction of the red box with G4 structures, isothermal titration calorimetry (ITC) experiments^45^ were performed using *c-MYC* and *c-KIT2*, the G4 structures showing the highest stabilization in the CD melting experiments, as well as h-DNA as a duplex DNA model. Measurements were conducted at both pH 6.3 (Fig. S17 and Table S4), where the acidic form of the red box, RH_2_^4+^, is the only species, and pH 7.2 (Fig. 3 and Table 1), which more closely reflects the nuclear pH of living cells. The ITC titration curves at both pH values were fitted using a multiple-sites binding model. Similar binding constants were obtained at pH 6.3 and pH 7.2, indicating that partial deprotonation of the red box has little effect on G4 recognition. The binding constants at pH 7.2 align well with the CD melting results, confirming that the red box (hereafter referred to as R, representing the RH_2_^4+^ and R^2+^ mixture in equilibrium at pH 7.2) exhibits significantly higher affinity for G4 structures than for the duplex DNA control. In the case of *c-MYC* (Fig. 3a), two distinct binding events were observed, each proceeding through a different binding mode. As shown in Table 1, the first event involves the binding of two R molecules to the G4 structure with a nanomolar binding constant, *K*_D1_ ≈ 7 nM, and is characterized by a favourable enthalpic (Δ*H*) and significantly favourable entropic (Δ*S*) contributions. This affinity falls within the range reported for high-affinity *c-MYC* G4 ligands,^33^ including pyridostatin,^46^ Phen-DC3,^47^ and tetraazaperopyrene derivatives.^48^ Notably, this affinity is up to one or two orders of magnitude higher than those previously reported for the Pd(ii)- and Pt(ii)-based metallacycles developed by our group,^24,25^ supporting the conclusion that strong and selective G4 recognition can be achieved without relying on transition-metal coordination. As previously reported for other ligands,^49^ such an entropically driven process, accompanied by a smaller enthalpic change, may be attributed to the binding of R in the grooves of the G4 structure and probably to the resulting displacement of ordered water molecules from the G4 surface. In contrast, the second binding event involves the association of seven additional R molecules with lower affinity, *K*_D2_ ≈ 1.5 µM, driven primarily by favourable enthalpic contributions and accompanied by a slight entropic penalty. This latter behaviour is consistent with end-stacking interactions of the ligand at the terminal G-tetrads and/or partial intercalation.^50^ Importantly, both events are characterized by negative Gibbs free energy values (Δ*G*), confirming the spontaneous association of R with *c-MYC*. A similar two-site binding profile was observed for *c-KIT2* (Fig. 3b), with nanomolar affinity for the first binding event (*K*_D1_ ≈ 1.0 nM) and micromolar affinity for the second binding event (*K*_D2_ ≈ 3.3 µM). Both binding events are characterized by favourable enthalpic and entropic contributions, resulting in negative Gibbs free energy values, indicative of spontaneous binding. In the case of h-DNA (Fig. 3c), the titration data were fitted to an independent binding model. In this case, two R molecules bind to the double stranded DNA in a single event with a micromolar binding constant of *K*_D_ ≈ 1.5 µM. This interaction is two to three orders of magnitude lower than that observed for the first binding events of *c-MYC* and *c-KIT2*, consistent with the marked preference of R for G4 structures over duplex DNA.^51^

**Table 1:** Thermodynamic parameters obtained from ITC measurements for the interaction of R with *c-MYC*, *c-KIT2*, and h-DNA at pH 7.2

	*c-MYC*		*c-KIT2*		h-DNA
Step	1	2	1	2	1
*n*	2.6 ± 0.1	6.8 ± 0.4	2.1 ± 0.3	4.3 ± 0.1	2.3 ± 0.1
*K*_a_ a	(1.4 ± 0.2) × 10^8^	(6.7 ± 0.5) × 10^5^	(9.5 ± 0.6) × 10^8^	(3.0 ± 0.2) × 10^5^	(8.9 ± 6.2) × 10^5^
*K*_D_ b	(7.0 ± 0.8) × 10^−9^	(1.5 ± 0.1) × 10^−6^	(1.0 ± 0.1) × 10^−9^	(3.3 ± 0.2) × 10^−6^	(1.5 ± 0.8) × 10^−6^
Δ*H*^c^	−18.5 ± 1.3	−36.6 ± 2.2	−18.6 ± 1.0	−29.1 ± 1.2	−22.6 ± 2.2
*T*Δ*S*^c^	28.0 ± 1.2	−3.3 ± 2.2	32.6 ± 1.0	2.2 ± 1.3	11.0 ± 3.9
Δ*G*^c^	−46.5 ± 0.3	−33.2 ± 0.2	−51.2 ± 0.2	−31.3 ± 0.7	−33.5 ± 1.7

a M^−1^.

b M.

c kJ mol^−1^.

Overall, the thermodynamic parameters obtained for *c-MYC* and *c-KIT2* suggest that the first binding event may involve groove recognition, as similarly entropically driven mechanisms have been reported for small molecules interacting within the grooves of G4 structures, while intercalation and end-stacking are typically enthalpically dominated.^49,50,52^ To further investigate this possibility, additional CD spectra were recorded. As shown in figure S13, addition of 3 equivalents of the red box to both *c-MYC* and *c-KIT2* resulted in the appearance of induced CD bands in the 350–400 nm region, providing additional experimental support for the proposed groove-binding contribution.

### The macrocyclic architecture of the red box is crucial for *c-MYC* stabilization

**Fig. 4:**
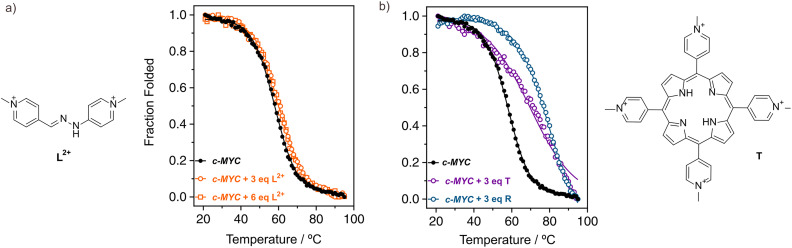
(a) CD melting curves at 263 nm of a 10 µM *c-MYC* solution in 10 mM lithium cacodylate, 1 mM KCl, 99 mM LiCl, pH 7.2 (LCB6), both in the absence (•) and in the presence of L^2+^ (3 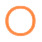 or 6 eq. 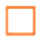). (b) CD melting curves at 263 nm of a 10 µM *c-MYC* solution in LCB6, both in the absence (•) and in the presence of TMPyP4 (T) (3 eq., 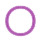) or R (3 eq., 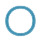).

Based on the observed stabilizing effect of R on the *c-MYC* structure and given the biological relevance of this proto-oncogene, further experiments were conducted to prove the structural features contributing to this interaction. To evaluate the importance of the macrocyclic topology, we compared the activity of R with that of its acyclic counterpart, L^2+^,^53^ which possesses the same pyridinium moieties. As shown in Fig. 4a, addition of 3 equivalents of L^2+^ induced only a modest stabilizing effect on *c-MYC* (Δ*T*_m_ = 2.9 ± 0.8 °C). Increasing the concentration to 6 equivalents, corresponding to the same number of pyridinium moieties present in 3 equivalents of R, resulted in only a marginal increase in stabilization (Δ*T*_m_ = 3.5 ± 2.0 °C), underscoring the critical role of the macrocyclic architecture for efficient G4 binding.

To further contextualize the role of the macrocyclic architecture of R as a selective G4 binder, we compared it to the well-characterized G4 binder TMPyP4, a porphyrin derivative known to bind *c-MYC* G4^44,54–56^ but also to interact with duplex DNA with similar affinities.^57^ Under identical experimental conditions, TMPyP4 produced a Δ*T*_m_ of 12.7 ± 1.4 °C, whereas R induced a higher stabilization (Δ*T*_m_ = 19.3 ± 0.6 °C), as shown in Fig. 4b, consistent with the high association constant determined by ITC. Overall, these findings suggest that the macrocyclic architecture of R is a key structural feature determining its strong affinity for G4 binding.

### The red box selectively binds the *c-MYC* G4 over model duplex DNA

To further assess the selectivity of R for *c-MYC* over duplex DNA, competitive binding assays were conducted using fluorescence resonance energy transfer melting competition (FRET-MC). In these experiments, the R*–c-MYC* complex was challenged with increasing concentrations of h-DNA and ds-DNA. The FAM-*c-MYC*-TAMRA (FMT) oligonucleotide was used as a fluorescently labelled analogue of the *c-MYC* sequence. Control CD melting experiments conducted with this labelled oligonucleotide in the presence of R confirmed that the 5′- and 3′-terminal fluorophores did not interfere with G4 folding or ligand induced stabilization, as comparable thermal stabilization was observed under the same experimental conditions as for the unlabelled counterpart (Fig. S14).

**Fig. 5:**
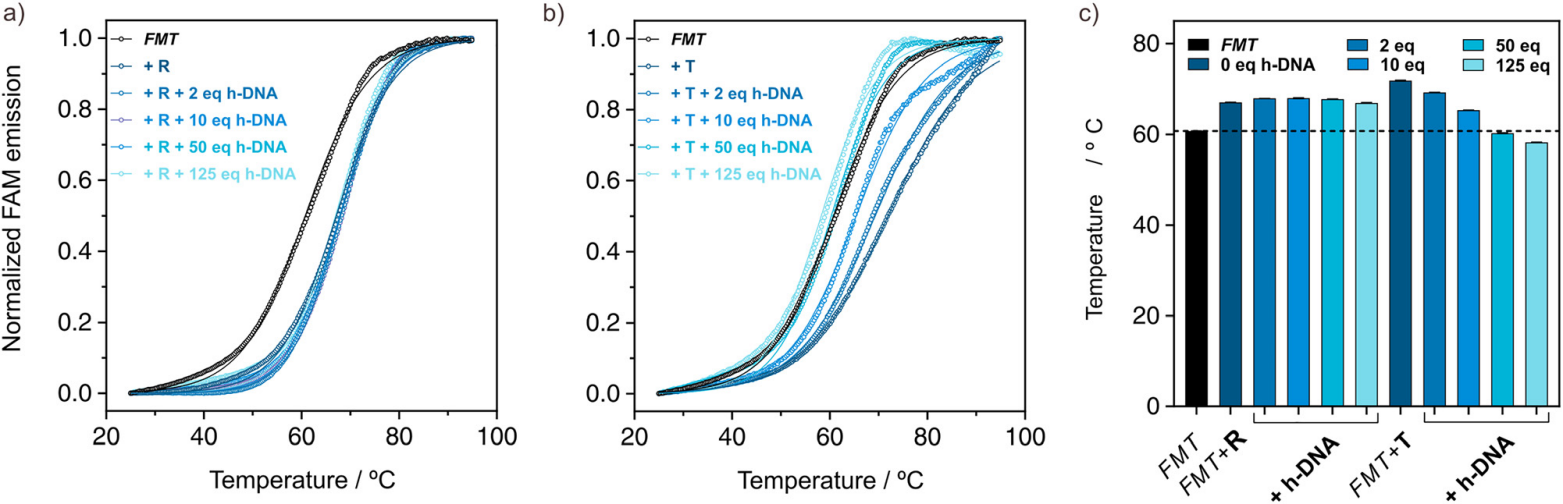
(a) Normalized FRET-melting profiles of 0.2 µM FAM-*c-MYC*-TAMRA G4 (FMT) in 10 mM lithium cacodylate, 10 mM KCl, 90 mM LiCl pH 7.2 (LCB5) (○), in the presence of 3 eq. of R (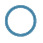), and after the addition of 2 (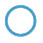), 10 (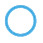), 50 (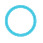) or 125 eq. (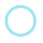) of h-DNA to the previous mixture.(b) Normalized FRET-melting profiles of 0.2 µM FMT in LCB5 (○), in the presence of 3 eq. of TMPyP4 (**T**) (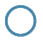), and after the addition of 2 (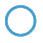), 10 (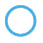), 50 (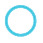) or 125 eq. (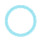) of h-DNA to the previous mixture. (c) Representation of the *T*_m_ (± SEM) of FMT alone and in the presence of R (3 eq.) or **T** (3 eq.), without and with h-DNA.

Melting curves were first recorded for the free FMT, followed by FMT in the presence of 3 equivalents of R, and subsequently for the resulting complex upon addition of increasing amounts of h-DNA or ds-DNA (2, 10, 50, and 125 eq.). A control FRET-MC experiment in which FMT was titrated with the same concentrations of h-DNA in the absence of the ligand showed negligible interaction between the duplex DNA and the G4 structure (Fig. S15). No decrease in the thermal stabilization of the *c-MYC* G4 was observed upon addition of up to 125 equivalents of the short h-DNA sequence (8 bp stem, 4T loop) to the R–G4 complex (Fig. 5a). In contrast, the longer ds-DNA sequence (20 bp), produced a slight reduction in G4 stabilization at 50 and 125 equivalents (Fig. S16). However, even under these strongly competitive conditions, the thermal stabilization conferred by R remained substantially higher than that of the free FMT, indicating that duplex DNA is unable to efficiently displace the ligand from the G4 structure. These results support a marked preference of R for G4 DNA over duplex DNA, consistent with the large difference in binding affinity determined by ITC. To further contextualize these findings, an identical FRET-MC experiment was performed using TMPyP4 as a control ligand (Fig. 5b). Consistent with its reported low selectivity and comparable affinities for duplex and G4 DNA (*K*_D_ ≈ 200 nM),^57^ progressive addition of h-DNA led to a continuous reduction of G4 thermal stabilization (Fig. 5c). The contrasting behaviour observed for TMPyP4 and R further highlights the strong preference of R for G4 structures.

### The red box downregulates the expression of *c-MYC* in cells

**Fig. 6:**
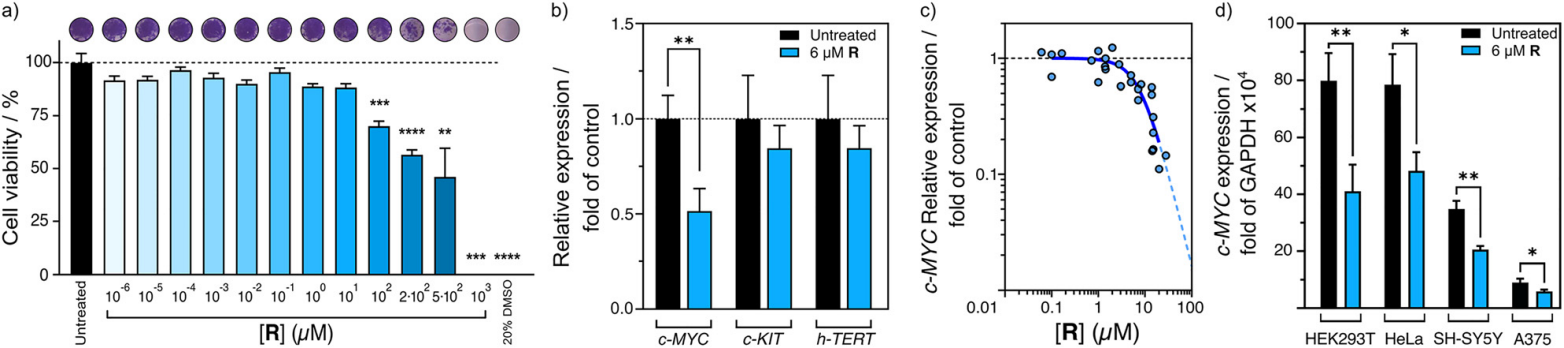
(a) Cell viability of HEK293T cells after treatment with different R concentrations for 72 h. (b) Relative gene expression levels of *c-MYC*, *c-KIT* and *hTERT* in HEK293T cells after treatment with 6 µM R in growth medium for 24 h. (c) Dose–response analysis of relative *c-MYC* mRNA expression in HEK293T cells treated with R (0.1–20 µM final concentration) for 24 h. Expression levels were normalized to untreated controls and fitted using a nonlinear regression model. (d) Relative *c-MYC* expression in HET293T, HeLa, SH-SY5Y, and A375 cells after treatment with 6 µM R in growth medium for 24 h, using GAPDH as the housekeeping gene. Data are presented relative to untreated controls and statistical analyses were performed using Kruskal-Wallis or Student's *t*-test, *P < 0.05, **P < 0.01, ***P < 0.001, ****P < 0.0001.

Considering the selective interaction of R with G4s (particularly *c-MYC* and *c-KIT2*) observed in the biophysical studies, we next evaluated whether this selectivity was preserved in a cellular context. Cytotoxicity was first assessed in HEK293T cells using crystal violet staining. As shown in Fig. 6a and Fig. S18, R displayed an IC_50_ value of 294.2 µM, while 10 µM was identified as the highest non-toxic concentration over a 72-hour incubation period. Therefore, all gene expression studies were performed at 6 µM, a concentration well below the cytotoxic threshold, to minimize indirect effects arising from cell death, reduced cell numbers, or other cellular stress responses.

RT-qPCR analyses were performed to determine whether R modulates gene expression at the mRNA level. HEK293T cells were selected as the experimental model because they express the target genes at detectable levels (see the SI, Fig. S19). Gene expression analyses were carried out for *c-MYC*, *c-KIT* and *hTERT*, the latter being a G4-containing gene that was also stabilized by R in CD melting assays. As shown in Fig. 6b, treatment with R resulted in a significant reduction in *c-MYC* mRNA levels, whereas no statistically significant changes were observed for *c-KIT* and *hTERT*. In the case of *c-KIT*, which contains multiple G4-forming regions, including *c-KIT1* and *c-KIT2*, the absence of a significant change in mRNA levels may be explained by the predominant regulatory role of *c-KIT1* over *c-KIT2*, as previously reported by Terenzi and co-workers.^58^

To further evaluate the cellular response, a dose–response experiment was performed. Treatment with increasing concentrations of R led to a progressive reduction in *c-MYC* mRNA levels, resulting in an approximately 90% decrease relative to untreated cells at the highest concentration tested (Fig. 6c). Finally, the effect of R on *c-MYC* expression was examined in three additional human cancer cell lines: SH-SY5Y (neuroblastoma), A375 (melanoma), and HeLa (cervical cancer). Cytotoxicity data for these cell lines are shown in Fig. S18. As shown in Fig. 6d, treatment with R under non-cytotoxic conditions resulted in significant *c-MYC* downregulation in all cells examined, with reductions ranging from approximately 35% for A375, 39% for HeLa, 41% for SH-SY5Y and 49% for HEK293T, despite the markedly different cytotoxicity profiles observed among them. Together, these results demonstrate that the ability of R to modulate *c-MYC* expression is not restricted to a particular cellular background and can be detected in both transformed and tumour-derived cell lines.

## Conclusions

We have demonstrated that the red box is the first fully organic member of the ExBox family of macrocyclic receptors capable of functioning as a G4 binder. This polycationic cyclophane exhibits a marked preference for binding the *c-MYC* and *c-KIT2* G4s over other G4 structures examined in this study, including telomeric and promoter G4 regions of relevant proto-oncogenes, as well as over duplex DNA models. Importantly, the red box displays nanomolar affinity for the *c-MYC* G4, with binding constants within the range reported for high-affinity *c-MYC* G4 ligands. In competition assays, the red box also showed higher selectivity for *c-MYC* over duplex DNA than the well-known macrocyclic porphyrin TMPyP4. Notably, the red box exhibited low cytotoxicity toward human cells, and treatment of HEK293T cells with non-cytotoxic concentrations resulted in selective downregulation of *c-MYC* expression, while the transcriptional levels of *c-KIT* and *hTERT* remained unaffected. Moreover, *c-MYC* downregulation was consistently observed across multiple cellular models, including both transformed and tumour-derived cell lines, supporting the biological relevance of the observed molecular interactions. Collectively, these results identify the red box as a metal-free macrocycle that combines nanomolar G4 affinity, G4-over-duplex selectivity exceeding that of TMPyP4, and functional *c-MYC* downregulation reproducible across four human cell lines, a combination of properties previously restricted to transition-metal-based metallacycles. This work therefore establishes hydrazone-based dynamic covalent chemistry as a viable, synthetically accessible design platform for translationally relevant G4-targeted gene regulation.

## Author contributions

P. N.: formal analysis, investigation, writing – original draft, and writing – review & editing; I. M. d. O.: formal analysis, investigation, writing – original draft, and writing – review & editing; C. G.-D.: formal analysis, investigation, and writing – review & editing; A. S.-T.: formal analysis, investigation, writing – original draft, and writing – review & editing; E. P.: conceptualization, funding acquisition, resources, supervision, writing – original draft, and writing – review & editing; C. P.: conceptualization, funding acquisition, resources, supervision, writing – original draft, and writing – review & editing.

## Conflicts of interest

There are no conflicts to declare.

## Supplementary Material

CB-OLF-D5CB00329F-s001

## Data Availability

The data supporting this article have been included in the article and in its supplementary information (SI). Supplementary information: materials and methods, analytical data, CD spectroscopy, ITC, and cell assays. See DOI: https://doi.org/10.1039/d5cb00329f.
