# Supplementary material for: The red box: a selective G-quadruplex binder that downregulates c-MYC expression in human cells
Source: RSC Chem Biol. 2026 Aug 18;7(9):1743–50. doi: 10.1039/d5cb00329f (PMC13483072; doi:10.1039/d5cb00329f)
Supplement: Supplementary file 1 [file CB-007-D5CB00329F-s001.pdf]

Supplementary Information (SI) for RSC Chemical Biology.
This journal is © The Royal Society of Chemistry 2026

SUPPORTING INFORMATION

The Red box as selective G-quadruplex binder that downregulates *c-MYC* expression in human cells

Paula Novo, [a,†] Iván Montes de Oca, [a,†] Claudia García-Delgado, [a] Agustín Sánchez-Temprano, [a] Elena Pazos [a,b]* and Carlos Peinador [a]*

[a] CICA – Centro Interdisciplinar de Química e Bioloxía and Departamento de Química, Facultade de Ciencias, Universidade da Coruña, A Coruña, 15071, Spain.

[b] Oportunius, Axencia Galega de Innovación, 15702 Santiago de Compostela, Spain.

* To whom correspondence should be addressed. Tel: +34 881015589/881015590; Email: elena.pazos@udc.es, carlos.peinador@udc.es

MATERIAL AND METHODS 2
CHARACTERIZATION OF THE RED BOX 8
PREPARATION OF THE FOLDING BUFFERS 9
CD SPECTROSCOPY 10
FRET MELTING COMPETITION ASSAYS 17
ITC EXPERIMENTS 19
CELL ASSAYS 20

MATERIAL AND METHODS

Chemicals

Unless otherwise specified, chemicals were purchased from *Fisher Scientific* and *Merck*. Water was purified using a Milli-Q system (*Millipore*).

Oligonucleotides

Oligonucleotides (sequences listed in Table 1) were purchased purified by RP-HPLC and lyophilized from *Biomers.net* (Ulm, Germany), and used without further purification. The dried oligonucleotides were dissolved in Milli-Q H_2O to prepare 500 μM stock solutions. Concentrations were determined by UV spectroscopy using a *Jasco* V-650 spectrophotometer using a Hellma 114-QS Semi-Micro cell (10 mm path length) at 25 ºC, using the molar extinction coefficients at 260 nm, as provided by the supplier: 257200 M^{-1} cm^{-1} for *h-TELO*, 323200 M^{-1} cm^{-1} for *c-MYC*, 261300 M^{-1} cm^{-1} for *c-KIT1*, 236300 M^{-1} cm^{-1} for c-*KIT2*, 483100 M^{-1} cm^{-1} for *hTERT*, 233000 M^{-1} cm^{-1} for the complementary strand of *c-MYC*, 368400 M^{-1} cm^{-1} for FAM-*c-MYC*-TAMRA (*FMT*), 212000 M^{-1} cm^{-1} for the h-DNA, and 205600 M^{-1} cm^{-1} and 217000 M^{-1} cm^{-1} for each of the single strands of the ds-DNA.

Table S1. List of the selected G4-forming and duplex oligonucleotides.

Oligo	Sequences (5′ → 3′)
c-MYC	TGGGGAGGGTGGGGAGGGTGGGGAAGG
FAM-*c-MYC*-TAMRA	FAM-TGGGGAGGGTGGGGAGGGTGGGGAAGG-TAMRA
c-KIT1	AGGGAGGGCGCTGGGAGGAGGG
c-KIT2	CGGGCGGGCGCGAGGGAGGGG
h-TELO	AGGGTTAGGGTTAGGGTTAGGG
hTERT	GGGGGCTGGGCCGGGGACCCGGGAGGGGTCGGGACGGGGCGGGG
BCL2	CGGGCGCGGGAGGAATTGGGCGGGAGC
VEGF	GGGGGCGGGCCGGGGGCGGGGTCCCGGCGGGGCGGAG
TERRA	UUAGGGUUAGGGUUAGGGUUAGGG
h-DNA	CGAATTCGTTTTCGAATTCG

ds-*c-MYC*	TGGGGAGGGTGGGGAGGGTGGGGAAGG<br>CCTTCCCCACCCTCCCCACCCTCCCCA
ds-DNA	ACGAGATGACGTCATCTCCA<br>TGGAGATGACGTCATCTCGT

Hybridization of the oligonucleotide sequences was carried out in the corresponding folding buffer at the desired final concentration (typically 10 μM). Samples were heated to 95 ºC for 5 min using an AccuBlock Digital Dry Bath from *Labnet* and then allowed to cool slowly to room temperature (RT). For ds-*c-MYC*, equimolar concentrations (10 μM) of the complementary strands were mixed in the folding buffer, heated to 90 ºC for 10 min and then allowed to slowly cool to RT.

Synthesis, HPLC purification and quantification of the Red box

Red box (**RH_2**·4TFA) was synthesized according to the protocol described in the literature [1]. RP-HPLC purification was performed in a *Jasco* LC-4000 series using an Aeris semipreparative column from *Phenomenex* (peptide XB-C18 stationary phase, 5 µm, 100 Å pore size, 250 × 10 mm). HPLC-MS grade solvents were used to prepare the eluents: A: 0.1% TFA in H_2O and B: 0.1% TFA in CH_3CN. Collected fractions were lyophilized and stored at −20 °C. The identity of the purified yellow solid was confirmed by ^{1}H NMR spectroscopy.[1]

RP-HPLC analyses of the purified fractions were carried out in a *Thermo Scientific* UltiMate 3000 coupled to a photodiode array (PDA) detector, using an Aeris analytical column from *Phenomenex* (peptide XB-C18 stationary phase, 3.6 µm, 100 Å pore size, 150 × 2.1 mm). The standard analytical HPLC method involved an initial isocratic section with 5% B over 2 min followed by a linear gradient from 5 → 95% B over 23 min.

RH_2·4TFA was dissolved in H_2O, and its concentration was determined by UV spectroscopy on a *Jasco* V-650 spectrometer using a standard Hellma 114-QS Semi-Micro cell (10 mm path length) at 25 ºC, considering the molar extinction coefficients described in the literature:[1] 62054 M^{-1} cm^{-1} at 365 nm for **RH_2**$^{4+}$ and 61092 M^{-1} cm^{-1} at 464 nm for its conjugated base **R**$^{2+}$.

Circular Dichroism (CD) spectroscopy

CD measurements were carried out on a *Jasco* J-185 CD-ORD spectropolarimeter equipped with a PTC-423S Peltier Thermostatted Single Cell Holder (Water Cooled), using a Hellma 110-QS Macro cell (2 mm path length). All measurements were conducted at 25 ºC. CD spectra were recorded over the 200−500 nm range with a data pitch of 0.5 nm and a bandwidth of 1.3 nm.

CD titration experiments. For CD titration experiments, aliquots of a 200 μM $\mathbf{RH_2}^{4+}$ solution in 10 mM potassium phosphate buffer, 88 mM KCl, pH 6.3 (PPB) were added to 0.4 mL of a 3 μM *c-MYC* solution in PPB. For the final five titration points a 4 mM $\mathbf{RH_2}^{4+}$ stock solution in Milli-Q water was used. All spectra were recorded with a scan speed of 200 nm/min and averaged over four accumulations.

CD melting experiments. In a typical CD melting experiment, 0.4 mL of a 10 μM oligonucleotide solution in the appropriate folding buffer, or the same solution with 3 equivalents of ligand ($\mathbf{RH_2}^{4+}$, TMPyP4, or $\mathbf{L}^{2+}$) was used. All CD melting profiles were recorded by heating from 21 to 95 ºC in 1 ºC intervals. Ellipticity was monitored at the characteristic wavelength of each oligonucleotide, with a response time of 4 s and a delay time of 5 s. CD spectra were also recorded at 21 ºC (initial), 95 ºC (final), and again after cooling back to 21 ºC.

Ellipticity data were converted to the folded fraction using Equation 1:

$$\text{Fraction folded} = \frac{\mathrm{CD}_\lambda - \mathrm{CD}_\lambda^{\min}}{\mathrm{CD}_\lambda^{\max} - \mathrm{CD}_\lambda^{\min}} \qquad (1)$$

where CD_λ is the ellipticity at each temperature, $\mathrm{CD}_\lambda^{\min}$ is the minimum ellipticity, and $\mathrm{CD}_\lambda^{\max}$ is the maximum ellipticity at the monitored wavelength.

Melting temperatures, T_m for the free oligonucleotide and T'_m for the oligonucleotide-ligand complex, were determined by fitting the melting curves to a Boltzmann sigmoidal function using GraphPad Prism 8.0. The ligand induced thermal stabilization, ΔT_m, were calculated using Equation 2:

$$\Delta T_m = T'_m - T_m \qquad (2)$$

Fluorescence Resonance Energy Transfer (FRET) melting competition assays

Fluorescence Resonance Energy Transfer Melting Competition (FRET-MC) assays were conducted in the Applied Biosystems® 7500 Real-Time PCR cycler equipped with a 6-carboxyfluorescein (FAM) filter and a 96-well plate. An analogue of the *c-MYC* sequence, named FAM-*c-MYC*-TAMRA (*FMT*), was used with 6-carboxyfluorescein (FAM) conjugated to the 5′-end and the quencher tetramethylrhodamine (TAMRA) to the 3′-end.

Samples were prepared in a total volume of 25 μL, using 10 mM lithium cacodylate buffer, 1 mM KCl, 99 mM LiCl, pH 7.2 (LCB6). Depending on the experimental condition, each sample contained: 0.2 μM *FMT*, 3 equivalents of ligand (**R** or TMPyP4), and 2, 10, 50, or 125 equivalents of h-DNA or ds-DNA. FAM fluorescence intensity was monitored throughout the experiment. Melting temperatures were determined by analysing the FRET-MC data using the same method applied for the CD melting experiments.

Isothermal Titration Calorimetry (ITC) experiments

All ITC experiments were carried out on a Nano-ITC calorimeter from *TA Instruments* at 25°C. The sample cell was loaded with 950 μL of a 10 μM solution of *c-MYC*, *c-KIT2* or h-DNA in the appropriate folding buffer, while the syringe containing 250 μL of a 700 μM $\mathbf{RH_2}^{4+}$ or **R** solution in the same buffer. Both the sample cell and syringe solutions were degassed for 30 min prior to the titration.

A typical titration consisted of an initial injection of 2.86 μL followed by 35 injections of 6.86 μL, for a total of 36 injections. Injections were spaced 200 s apart, and the stirring speed was set to 250 rpm. Data were analysed using NanoAnalyze software by fitting to the appropriate binding model. Thermodynamic parameters (K_D, ΔH and ΔS) were determined with 5000 trials at a 95% confidence level. Baseline correction was performed by subtracting the heat of dilution, corresponding to the ligand’s contribution, from the final titration data.

Cell assays

Cell culture. Human embryonic kidney cells (HEK293T; CRL-3216, *ATCC*, Rockville, MD, USA) HeLa (CCL-2, *ATCC*, Rockville, MD, USA), SH-SY5Y (CRL-2266, *ATCC*, Rockville, MD, USA), and A375 (CRL-1619, *ATCC*, Rockville, MD, USA) cells were cultured in Dulbecco's Modified Eagle Medium (DMEM; *Corning*, NY, USA) supplemented with 10% fetal bovine serum (FBS; *Cytiva*,

MA, USA), and antibiotics (100 U/mL penicillin and 100 U/mL streptomycin; Lonza, Basel, Switzerland). Cells were maintained at 37 ºC in humidified atmosphere containing 5% CO_2.

Cytotoxicity assays. HEK293T, HeLa, SH-SY5Y or A375 cells were seeded into 96-well microplates at a density of 1.5×10^4 cells/cm^2 and cultured in growth medium for at least 18 hours. The following day, the medium was replaced with fresh growth medium containing increasing concentrations of **R** (ranging from 1 pM to 1 mM, , or to 4 mM for HeLA), and cells were incubated for 72 hours. Untreated cells were used as a negative control, while 20% DMSO in growth medium was used as a positive control for cytotoxicity.

Cell viability was assessed using crystal violet staining. Briefly, cells were fixed with methanol for 5 minutes at RT, washed with PBS, and stained with ready-to-use crystal violet solution (*Prolab Diagnostics*, Richmond Hill, ON, Canada) for 5 minutes at RT. Excess dye was removed by carefully washing three times with distilled water. Images of the stained wells were taken prior to solubilization of the dye with 10% (v/v) acetic acid. Absorbance was measured at 590 nm using a Synergy HTX plate reader (*Agilent*, CA, USA). Cell viability was determined by comparing the absorbance values of treated samples to those of the untreated control. All experiments were performed at least in triplicate to ensure reproducibility.

RNA level analyses. HEK293T cells were selected for gene expression experiments based on their endogenous expression levels of the *c-KIT*, *c-MYC*, and *hTERT*. Analysis of transcript expression data from the Human Protein Atlas revealed that HEK293T cells exhibit substantially higher basal expression of *c-KIT* than commonly used cancer cell lines such as HeLa and HepG2. In addition, HEK293T cells display robust *c-MYC* expression together with detectable *hTERT* expression. These expression levels provide a suitable model for evaluating the ability of Red box to modulate their transcription. Moreover, the use of a single cell line expressing all three targets enabled direct comparison of gene expression changes under identical experimental conditions while minimizing variability associated with the use of multiple cellular models.

Total RNA was extracted from near-confluent HEK293T, A375, SH-SY5Y or HeLa cells using NZYol (*Nzytech*, Lisbon, Portugal) according to the manufacturer's protocol. cDNA synthesis and quantification of *c-KIT*, *c-MYC* and *hTERT* expression levels were performed using the One-step NZYSpeedy RT-qPCR Green kit (*Nzytech*, Lisbon, Portugal) on a StepOnePlus Real-Time PCR System (*Applied Biosystems*, Waltham, MA, USA).

Each reaction mixture (10 µl total volume) contained 5 µl of 2× Reaction Mix, 0.4 µl of each forward and reverse primer (Table S2, 10 µM), 0.4 µl of One-Step RT-qPCR Enzyme Mix, 10 ng of total RNA, and nuclease-free water as required.

Table S2. List of the forward and reverse primers used for the RNA level analysis.

Oligo	Forward Primer (5′ → 3′)	Reverse Primer (5′ → 3′)
c-MYC	CCTGGTGCTCCATGAGGAGAC	CAGACTCTGACCTTTTGCCAGG
c-KIT	CACCGAAGGAGGCACTTACACA	TGCCATTCACGAGCCTGTCGTA
hTERT	GCCGATTGTGAACATGGACTACG	GCTCGTAGTTGAGCACGCTGAA
GADPH	GTCTCCTCTGACTTCAACAGCG	ACCACCCTGTTGCTGTAGCCAA

Samples were divided into two groups: a control group and a treatment group, in which 100 µL of an **R** solution were added to 1 mL of growth medium (final concentration 6 µM). Both groups were incubated for 24 hours at 37 ºC and in a 5% CO_2 atmosphere. A minimum of six biological replicates were included per group, with two technical replicates per sample. For dose-response experiments, samples were treated with increasing concentrations of an **R** solution (final concentrations ranging from 0.1 to 20 µM) and incubated under identical conditions. A minimum of three biological replicates were included per concentration, each with two technical replicates. Gene expression levels were normalized to GAPDH as an internal control, and relative expression was analysed using the comparative Ct method.[2]

Statistical analyses. All data are expressed as mean ± standard error of the mean (SEM). Outliers were identified using the ROUT test with a Q value of 5%. The normality of each dataset was assessed using the Kolmogorov-Smirnov test with Dallal-Wilkinson-Lilliefors P-value. An unpaired t-test was used to evaluate statistical significance between two independent datasets. For comparisons among more than two datasets, the Kruskal-Wallis test followed by Dunn's multiple comparison test (both uncorrected) was applied. Statistical significance was considered at $P<0.05$ and indicated with (*). (**), (***), (****) denote $P<0.01$, $P<0.001$, and $P<0.0001$, respectively.

CHARACTERIZATION OF THE RED BOX

RH₂·4TFA: RP-HPLC: t_R = 9.1 min. HR-MS (ESI): m/z $[M]^{2+}$ calcd for $C_{38}H_{34}N_8^{2+}$ = 301.1448; found: 301.1439 $[M]^{2+}$, 715.2737 $[M + TFA]^{+}$.

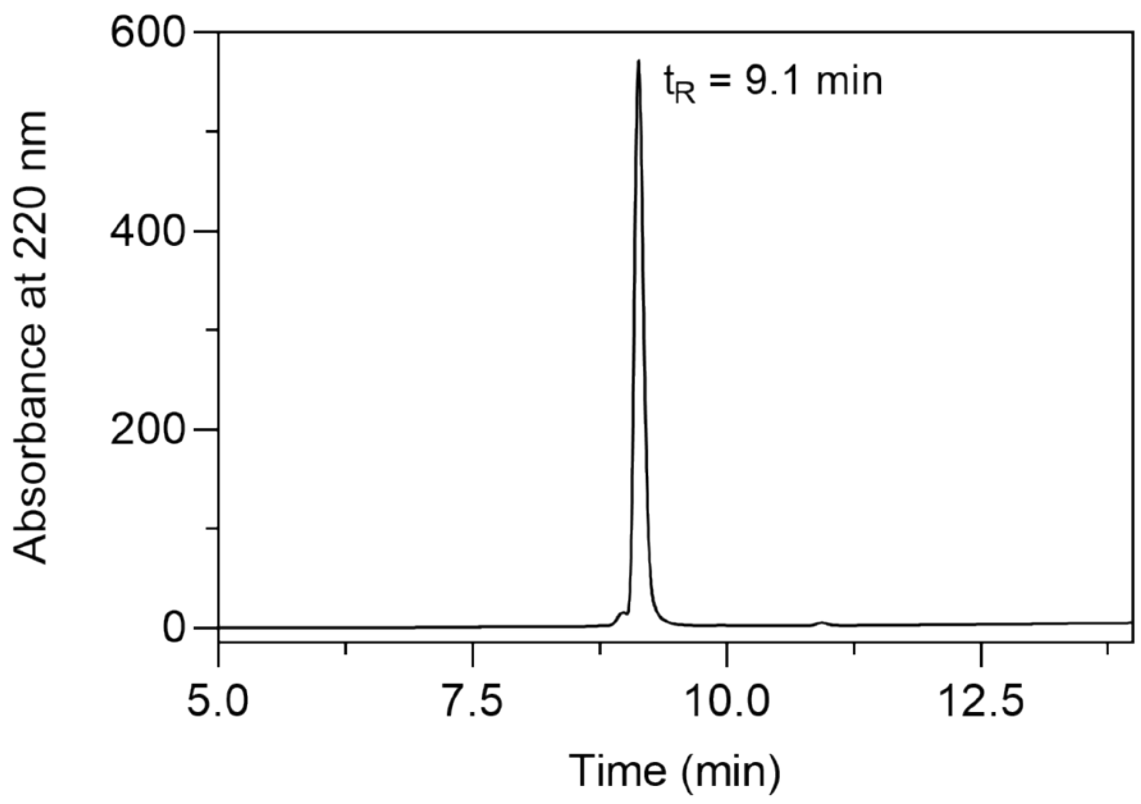

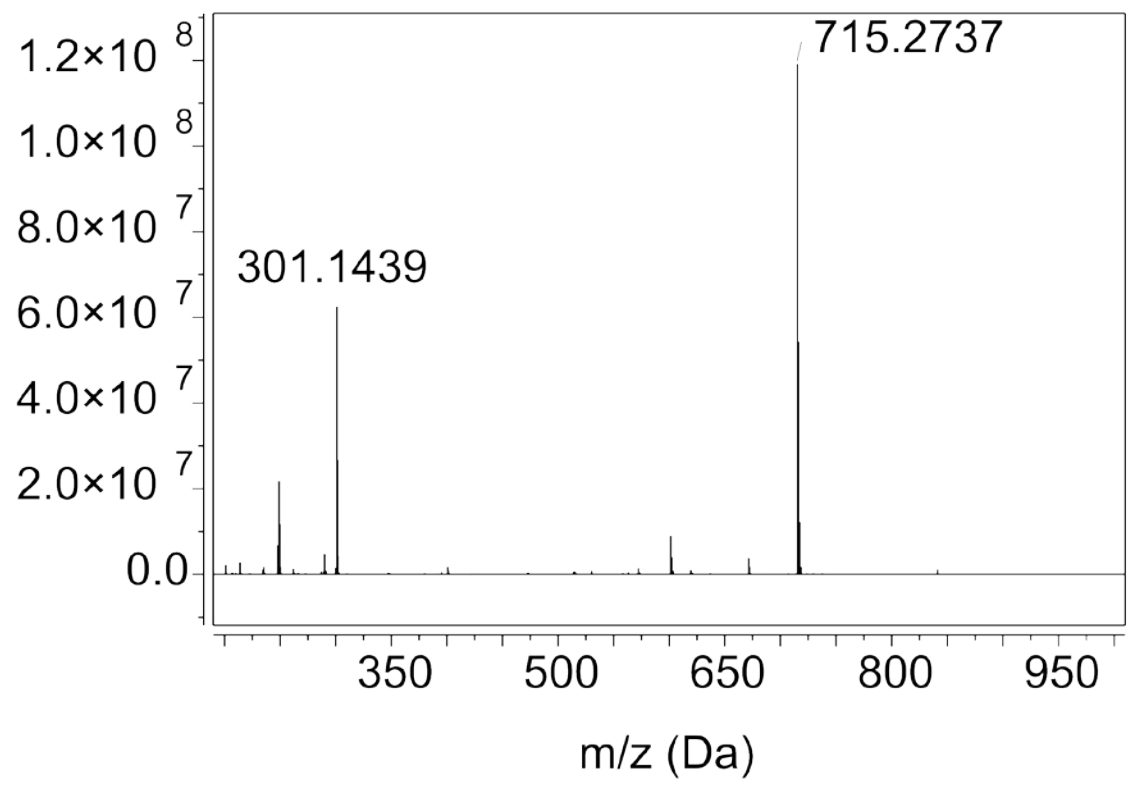

^{1}H NMR (300 MHz, D_2O, 300 K) δ: 8.76 (d, *J* = 6.7 Hz, 4H), 8.59 (broad s, 2H), 8.26 (s, 2H), 8.24 (d, *J* = 6.6 Hz, 4H), 7.69 (broad s, 2H), 7.72 (broad s, 2H), 7.62 (s, 4H), 7.54 (s, 4H), 7.24 (broad s, 2H), 5.82 (s, 4H), 5.54 (s, 4H).

The recorded NMR spectrum is in good agreement with that reported in the literature.[1] It reflects the typical coalescence and non-equivalence of the protons H_c-$H_{c'}$ and H_d-$H_{d'}$, on the upper and lower sides of the pyridinium moieties, due to the restricted rotation around the NH-C*sp*^{2} bond, which is the result of the highly delocalized nature of the π system.

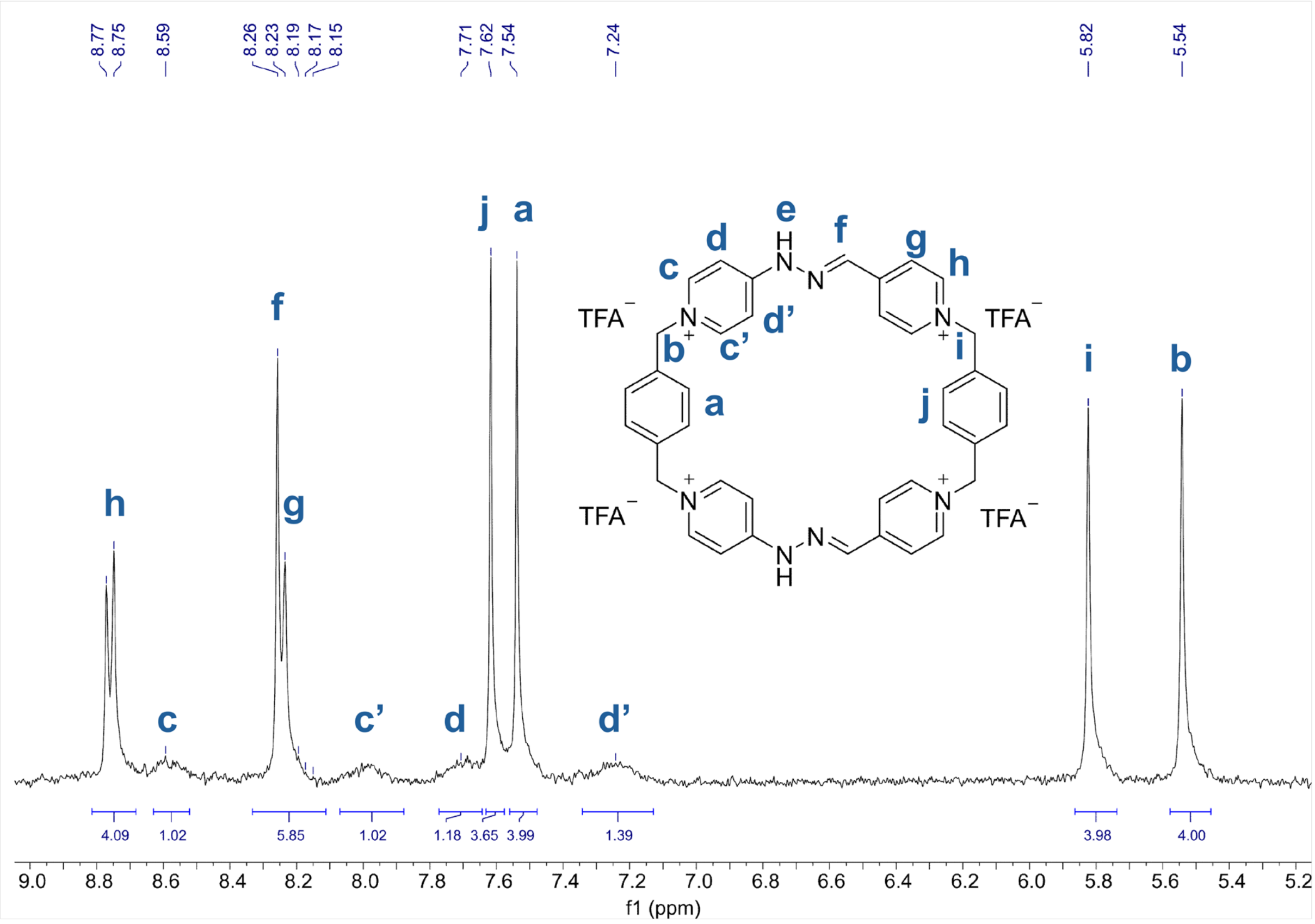

PREPARATION OF THE FOLDING BUFFERS

Potassium phosphate buffer (PPB) was prepared by mixing the corresponding volumes of 0.2 M K_2HPO_4 and 0.2 M KH_2PO_4 stock solutions, and the corresponding volume of a 2 M KCl stock solution to obtain a 10 mM PPB, 88 mM KCl, pH 6.3.

Lithium cacodylate buffers (LCB1 to LCB6) were prepared by diluting a 0.1 M cacodylic acid stock (stored at 4 ºC), together with the corresponding volumes of 1 M KCl and 1 M LiCl solutions, to obtain the desired concentrations. pH was adjusted to 6.3 or 7.2 by adding aliquots of a 1 M LiOH solution (taking advantage of the inert character of the Li^+ cation with respect to its negligible influence in the topology of the G4 structure).

Table S3. List of the different buffers used.

Buffer code	Buffer composition	pH
PPB	10 mM potassium phosphate, 88 mM KCl	6.3
LCB1	10 mM lithium cacodylate, 90 mM KCl, 10 mM LiCl	6.3
LCB2	10 mM lithium cacodylate, 10 mM KCl, 90 mM LiCl	6.3
LCB3	10 mM lithium cacodylate, 1 mM KCl, 99 mM LiCl	6.3
LCB4	10 mM lithium cacodylate, 100 mM LiCl	6.3
LCB5	10 mM lithium cacodylate, 10 mM KCl, 90 mM LiCl	7.2
LCB6	10 mM lithium cacodylate, 1 mM KCl, 99 mM LiCl	7.2

CD SPECTROSCOPY

Red box Spectra

CD spectra of a range of $\mathbf{RH_2}^{4+}$ concentrations were recorded. These solutions were prepared by the addition of the corresponding volumes of a 160 µM $\mathbf{RH_2}^{4+}$ stock solution in 10 mM potassium phosphate, 88 mM KCl, pH 6.3 (PPB).

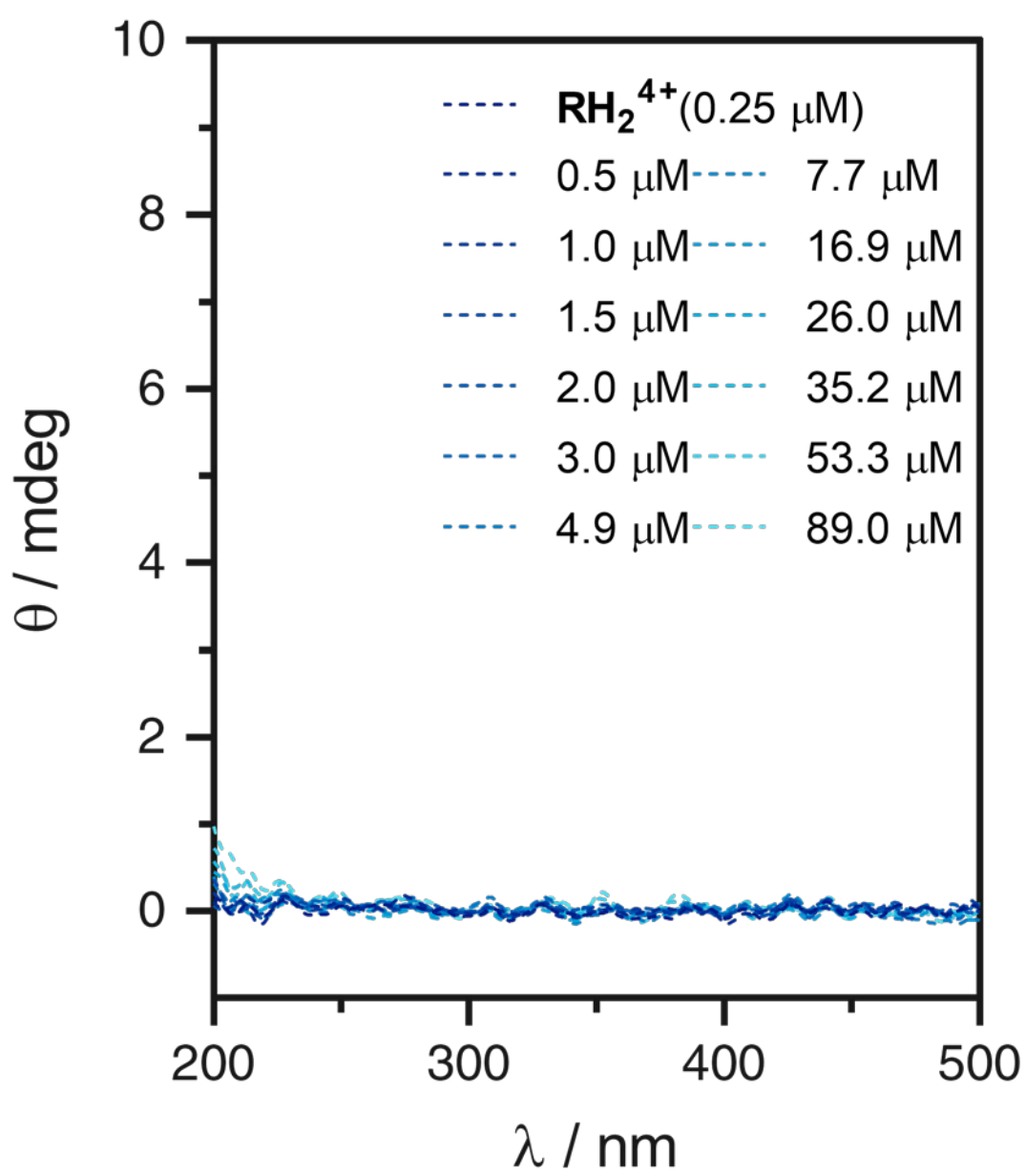

Figure S1. CD spectra of increasing $\mathbf{RH_2}^{4+}$ concentrations in PPB.

c-MYC spectra in phosphate and cacodylate buffers

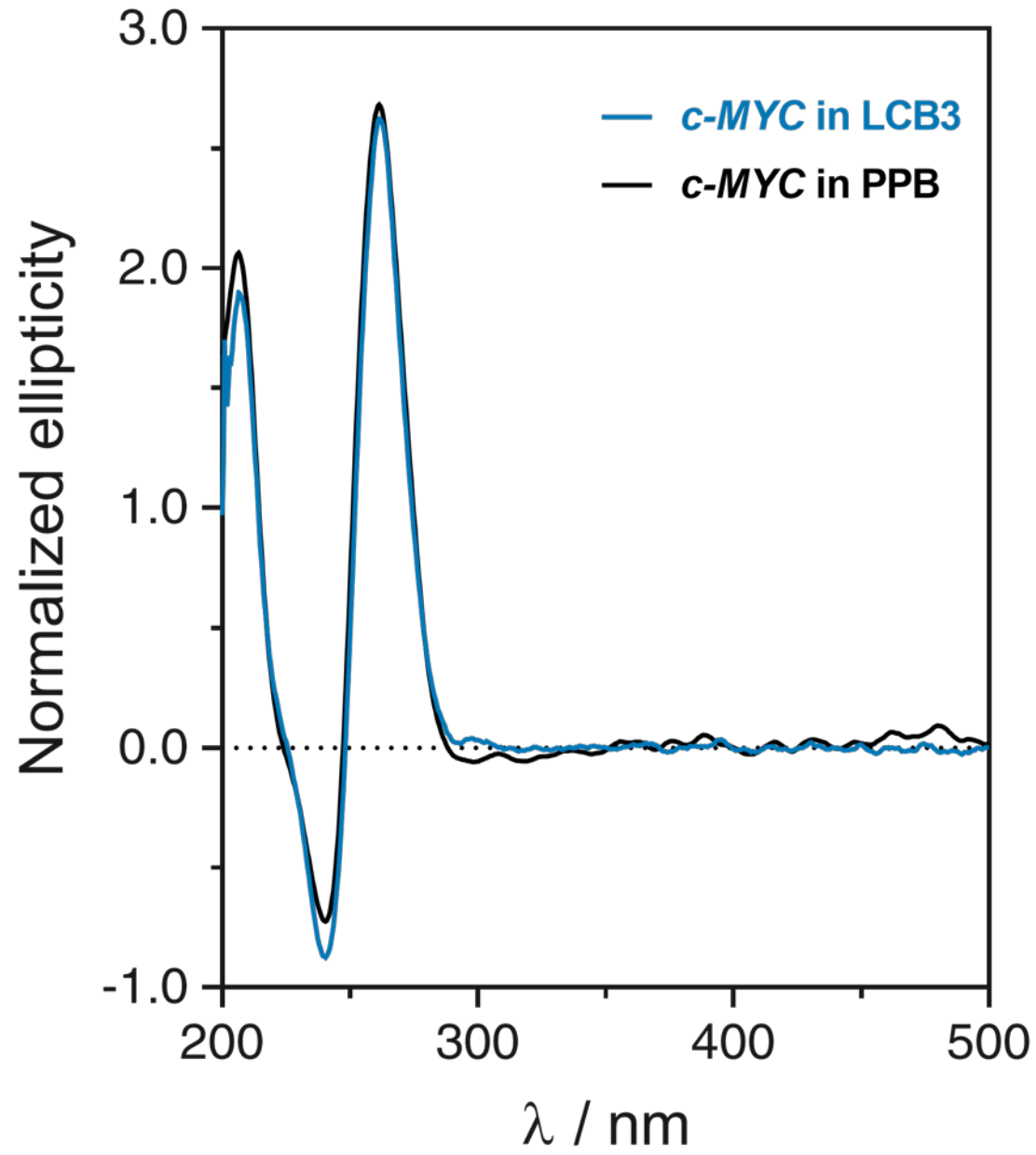

Figure S2. Normalized CD spectra of *c-MYC* solutions in PPB and LCB3 **at 25 °C.**

Red box interaction with G4s

RH_2^{4+} interaction with *c-KIT1*

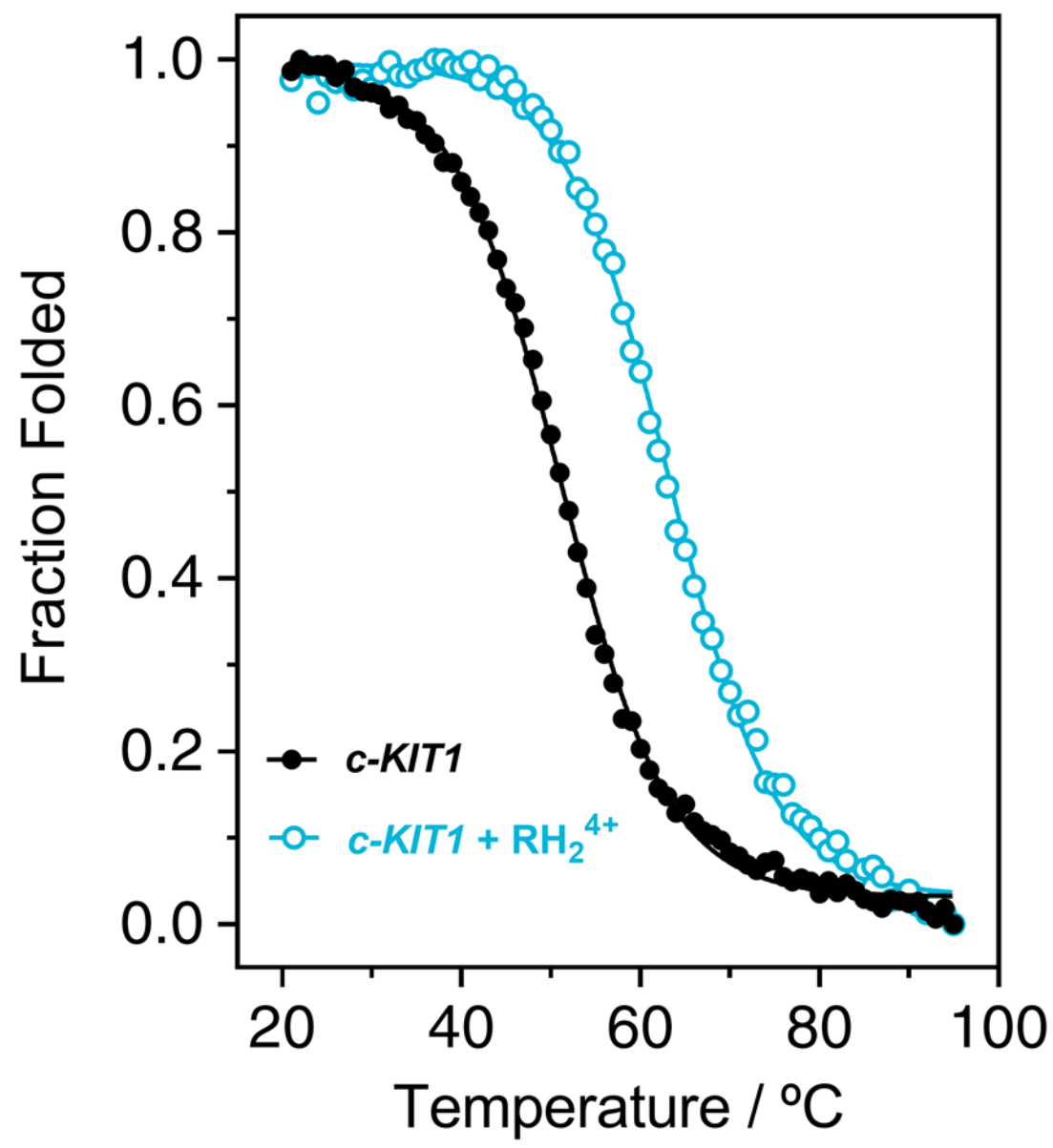

Figure S3. CD melting curves at 263 nm of a 10 µM *c-KIT1* solution in LCB1 in the absence (●) and in the presence of **RH_2^{4+}** (3 eq, ○).

RH_2^{4+} interaction with *c-KIT2*

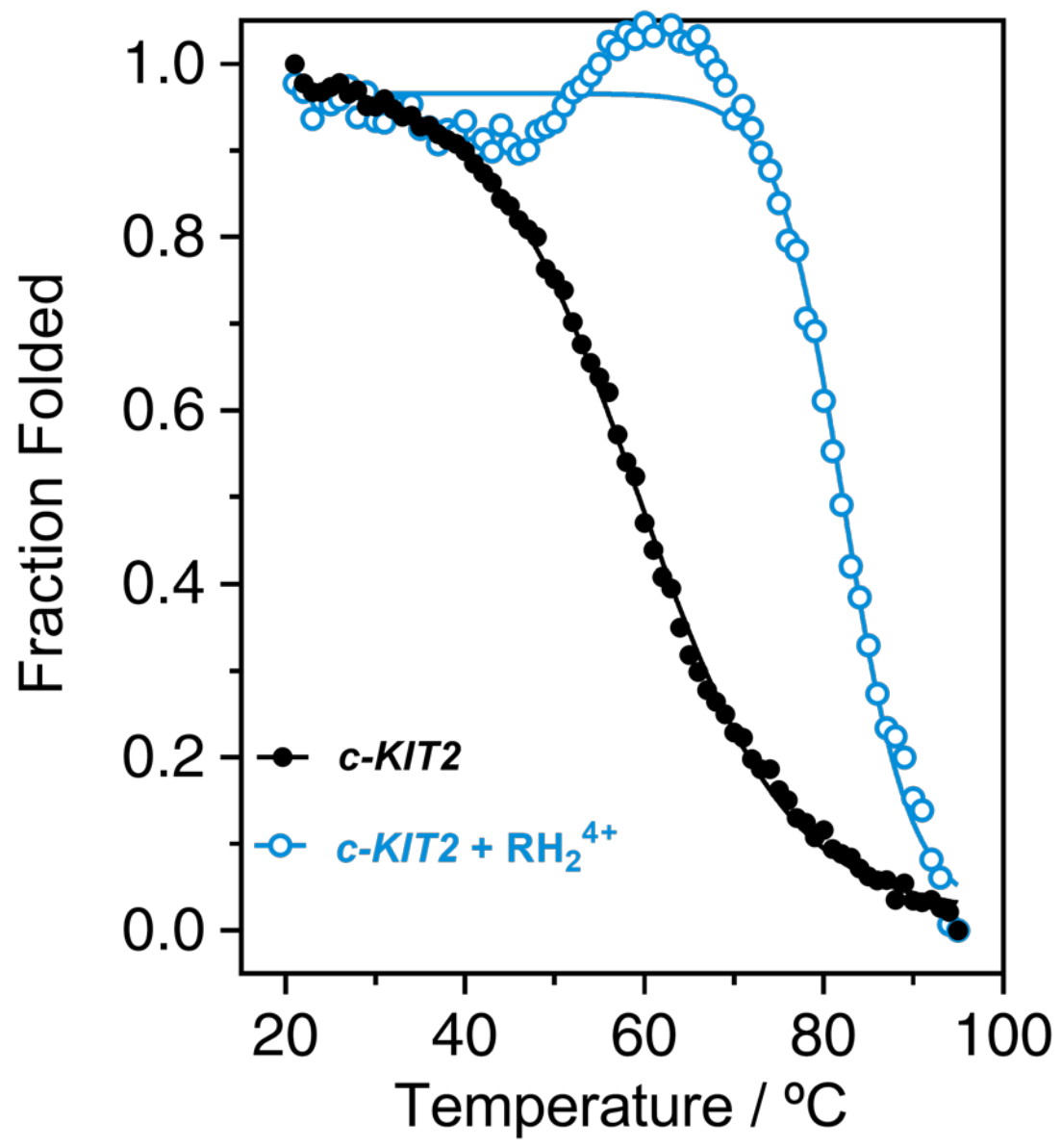

Figure S4. CD melting curves at 263 nm of a 10 μM *c-KIT2* solution in LCB2 in the absence (●) and in the presence of $\mathbf{RH_2^{4+}}$ (3 eq, ○).

RH_2^{4+} interaction with *hTERT*

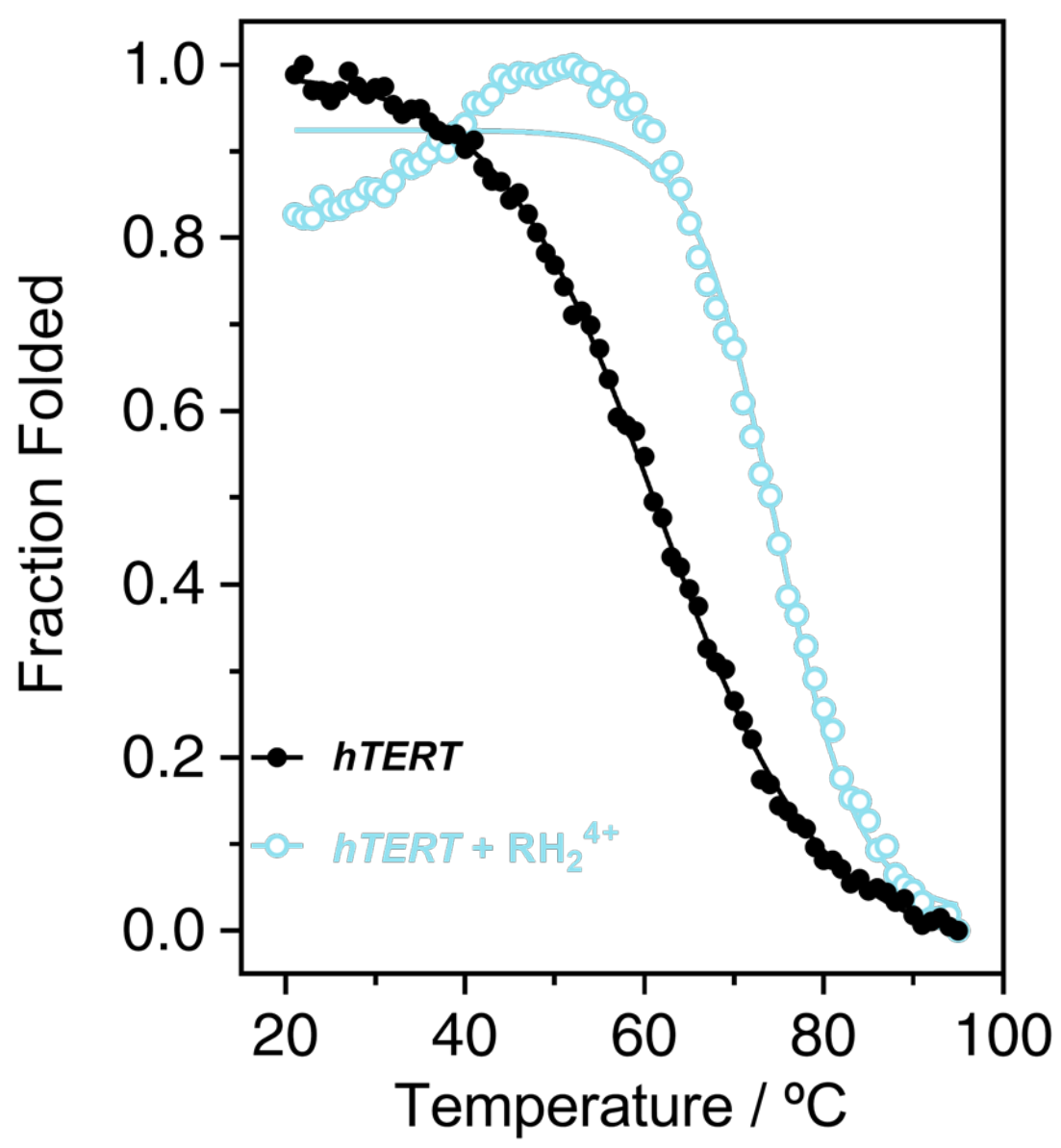

Figure S5. CD melting curves at 263 nm of a 10 μM *hTERT* solution in LCB3 in the absence (●) and in the presence of $\mathbf{RH_2^{4+}}$ (3 eq, ○).

RH_2^{4+} interaction with *h-TELO*

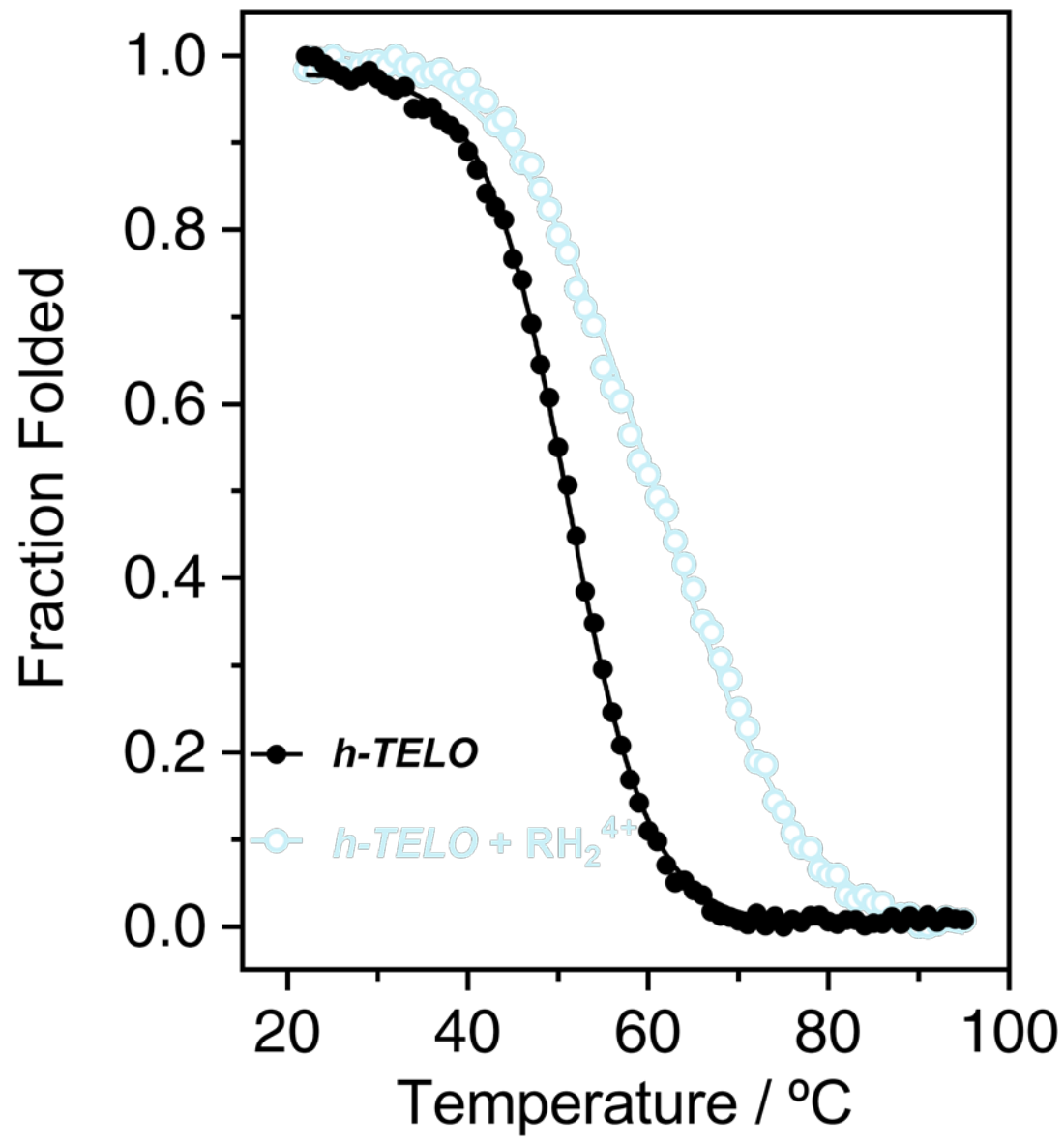

Figure S6. CD melting curves at 293 nm of a 10 µM *h-TELO* solution in LCB1 in the absence (●) and in the presence of $\mathbf{RH_2}^{4+}$ (3 eq, ○).

RH_2^{4+} interaction with *BCL2*

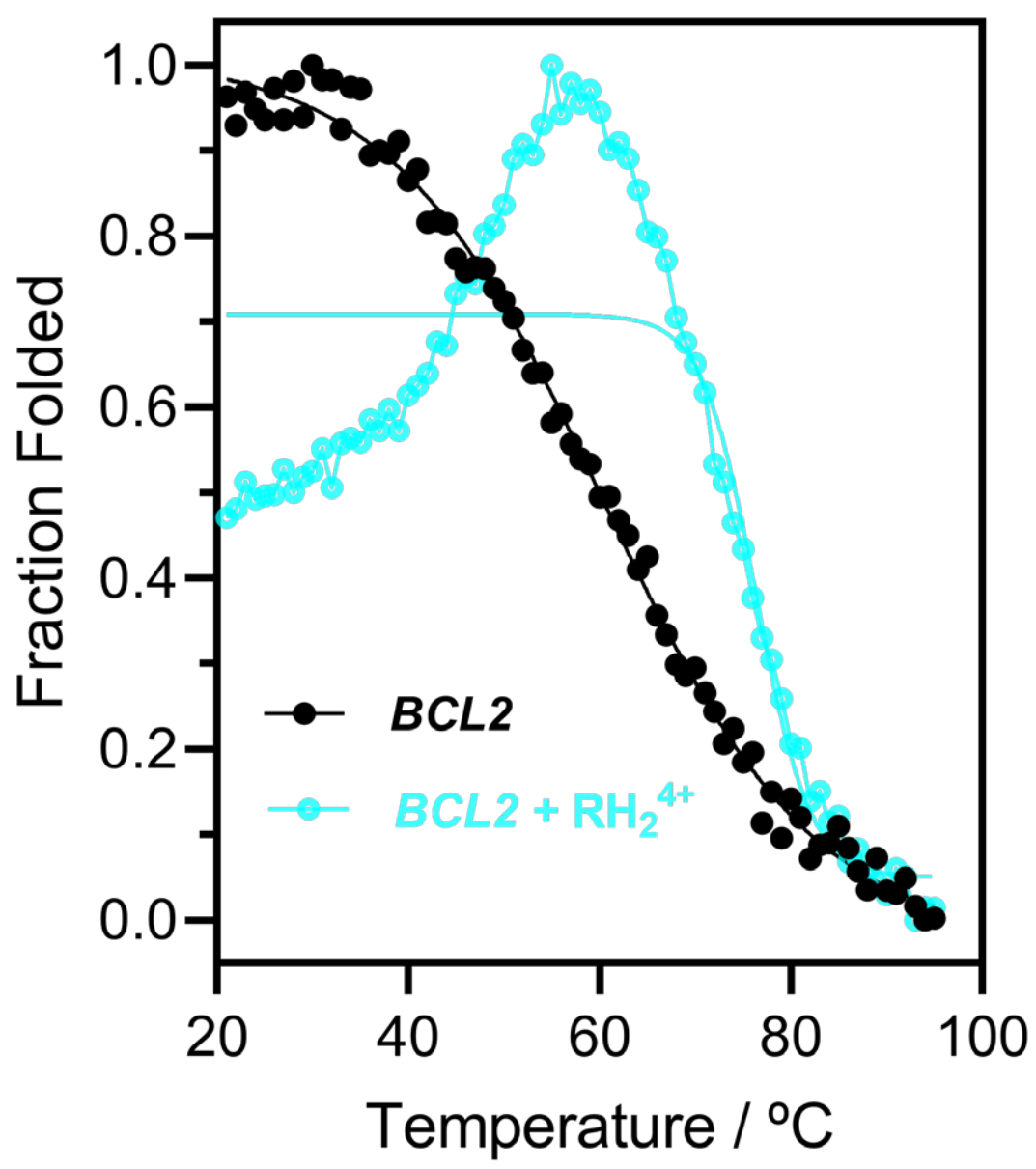

Figure S7. CD melting curves at 265 nm of a 10 µM *BCL2* solution in LCB2 in the absence (●) and in the presence of $\mathbf{RH_2}^{4+}$ (3 eq, ○).

RH_2^{4+} interaction with *VGEF*

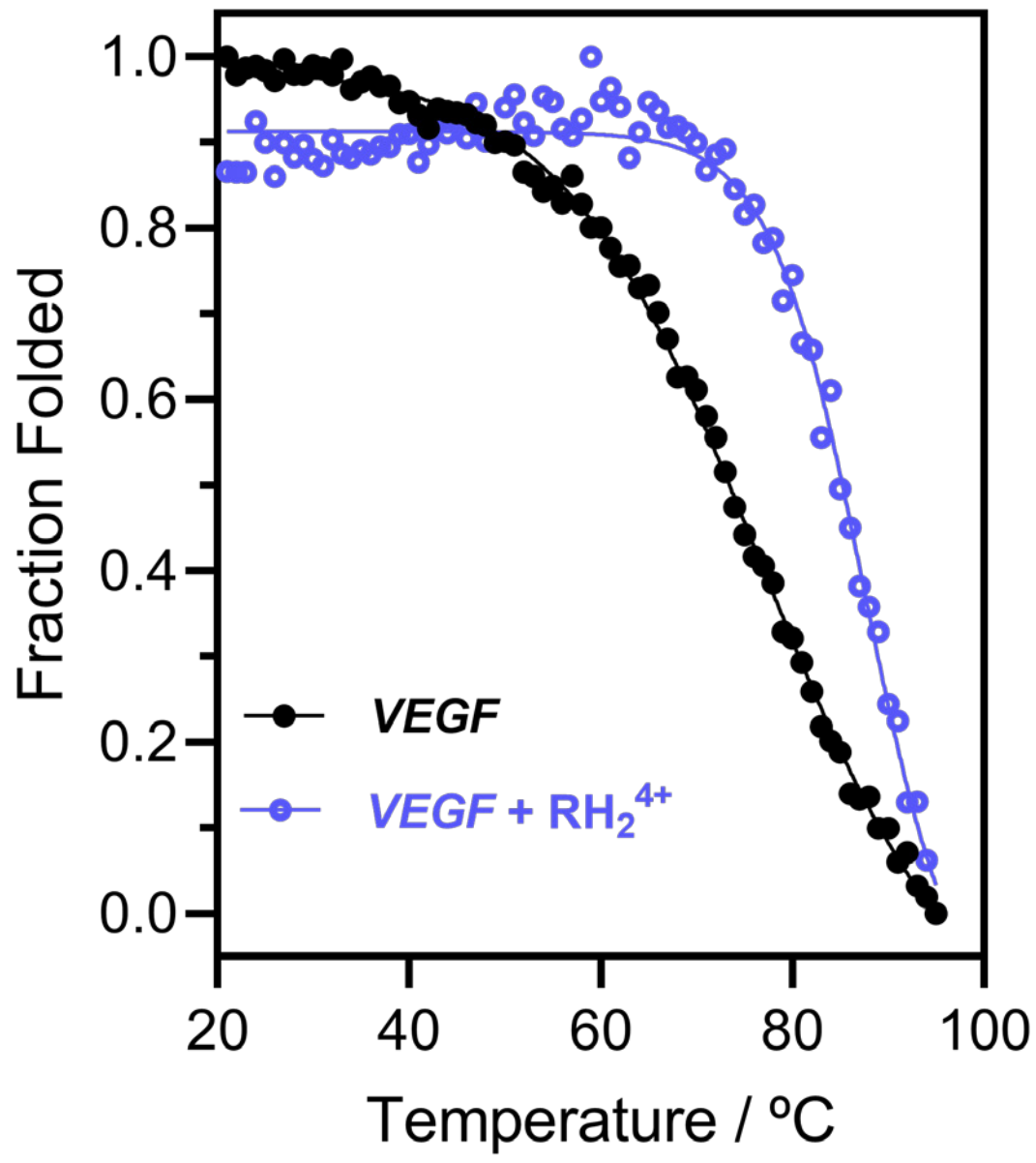

Figure S8. CD melting curves at 261 nm of a 10 µM *VEGF* solution in LCB3 in the absence (●) and in the presence of **RH_2^{4+}** (3 eq, ⦵).

RH_2^{4+} interaction with *TERRA*

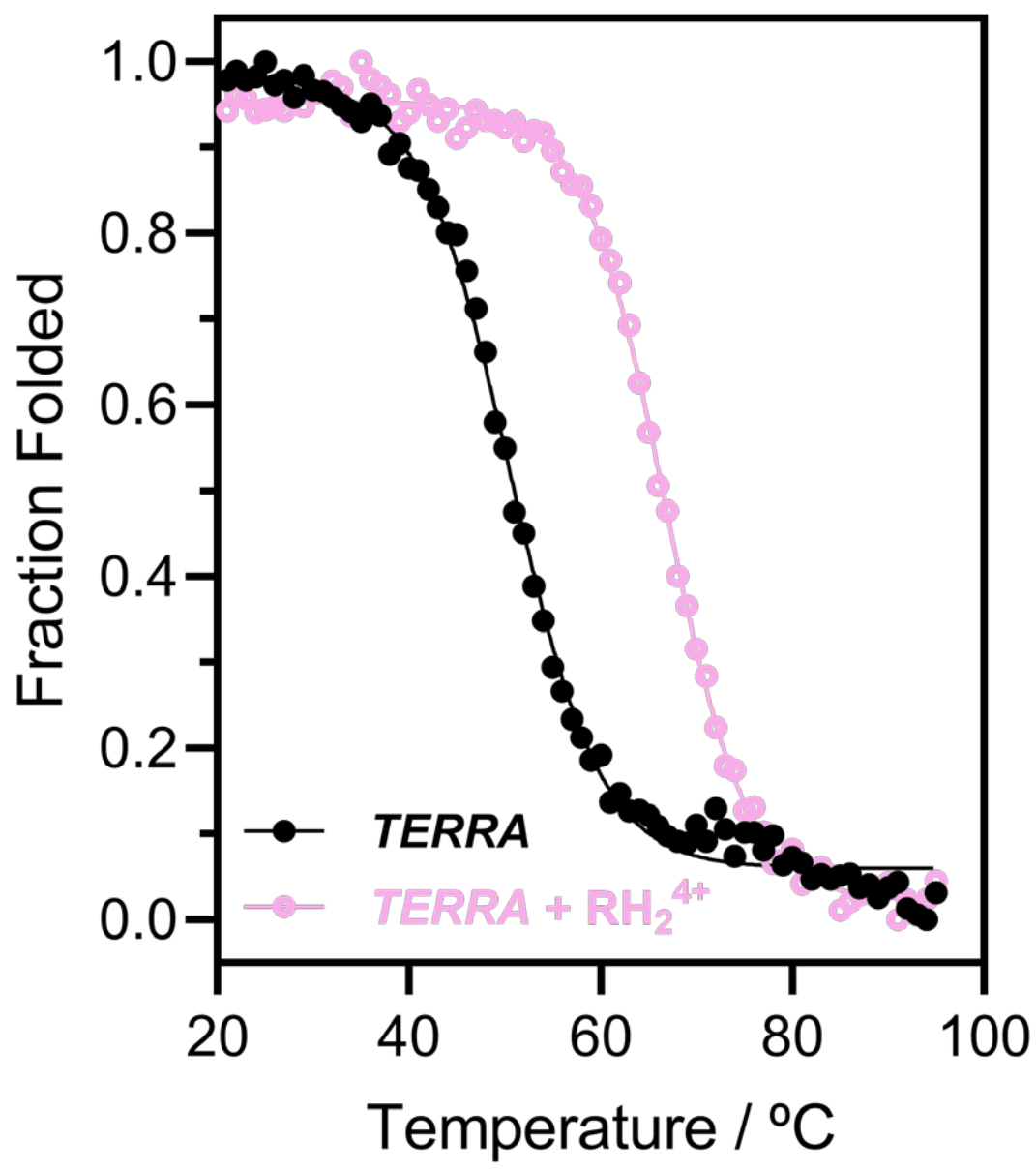

Figure S9. CD melting curves at 264 nm of a 10 µM *TERRA* solution in LCB3 in the absence (●) and in the presence of **RH_2^{4+}** (3 eq, ⦵).

Red box interaction with Duplex DNA

RH_2^{4+} interaction with ds-*c-MYC*

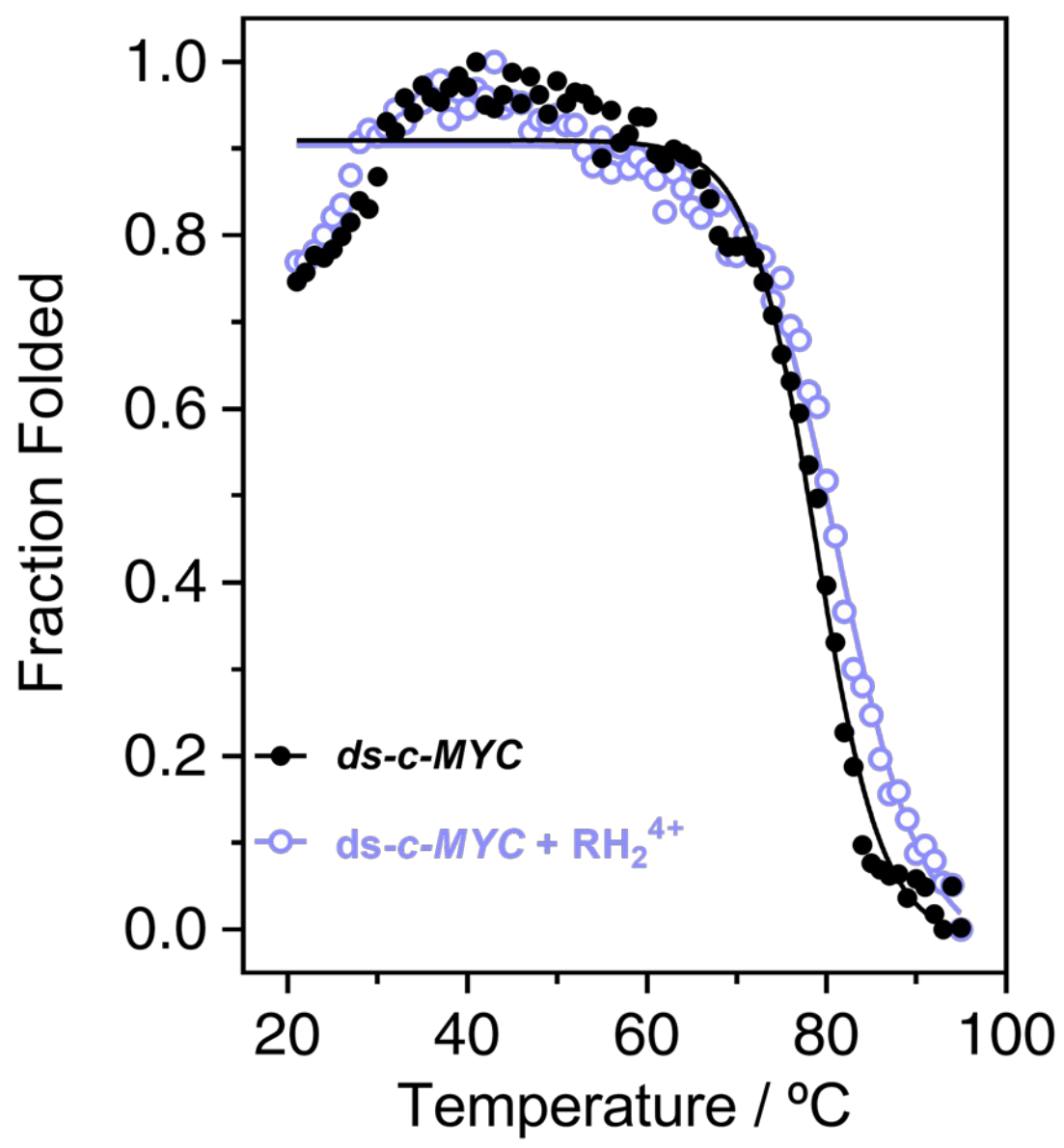

Figure S10. CD melting curves at 263 nm of a 10 µM ds-*c-MYC* solution in LCB3, in the absence (●) and in the presence of **RH_2^{4+}** (3 eq, ⊖).

RH_2^{4+} interaction with h-DNA

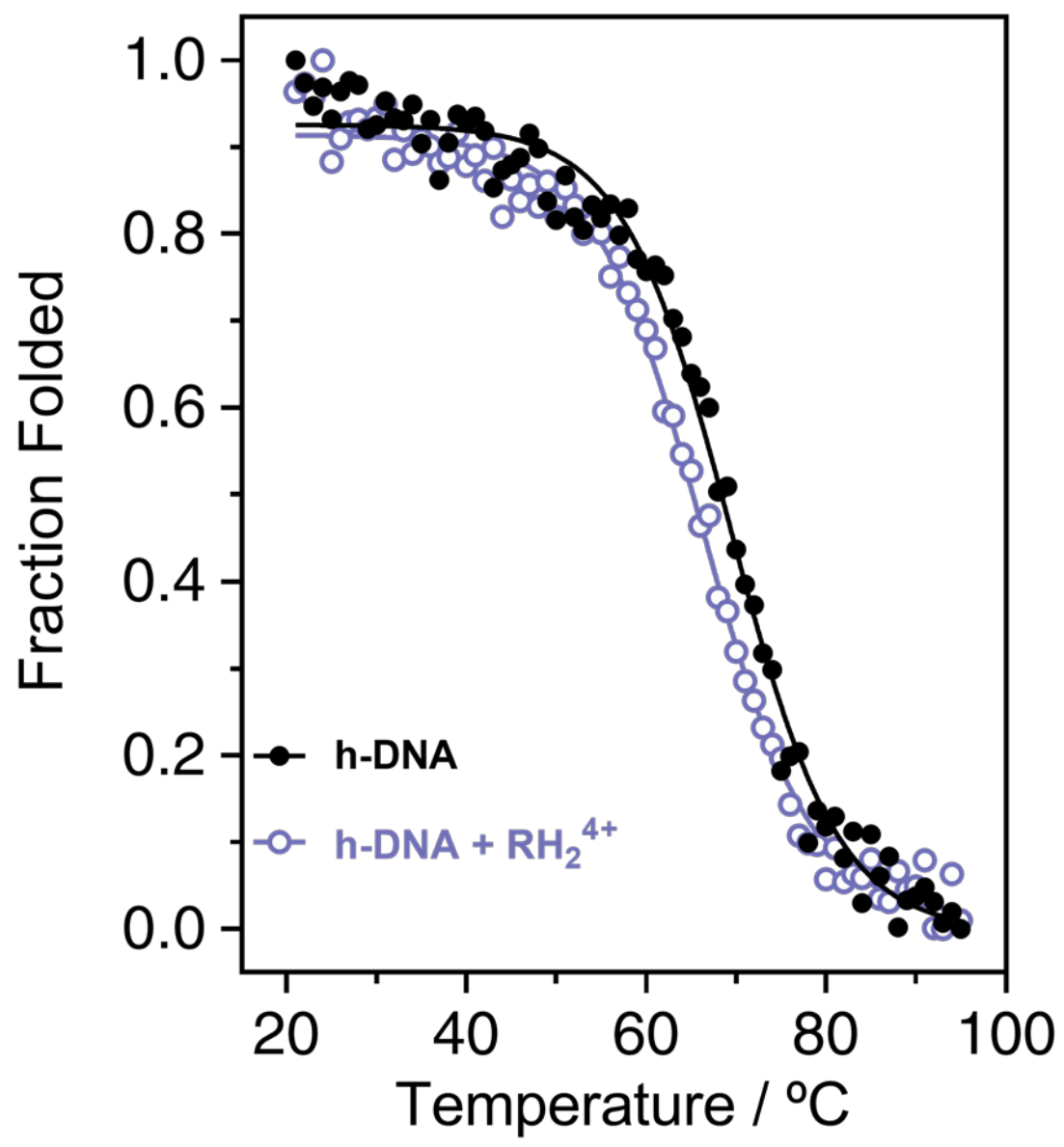

Figure S11. CD melting curves at 250 nm of a 10 µM h-DNA solution in LCB1, in the absence (●) and in the presence of **RH_2^{4+}** (3 eq, ⊖).

RH_2^{4+} interaction with ds-DNA

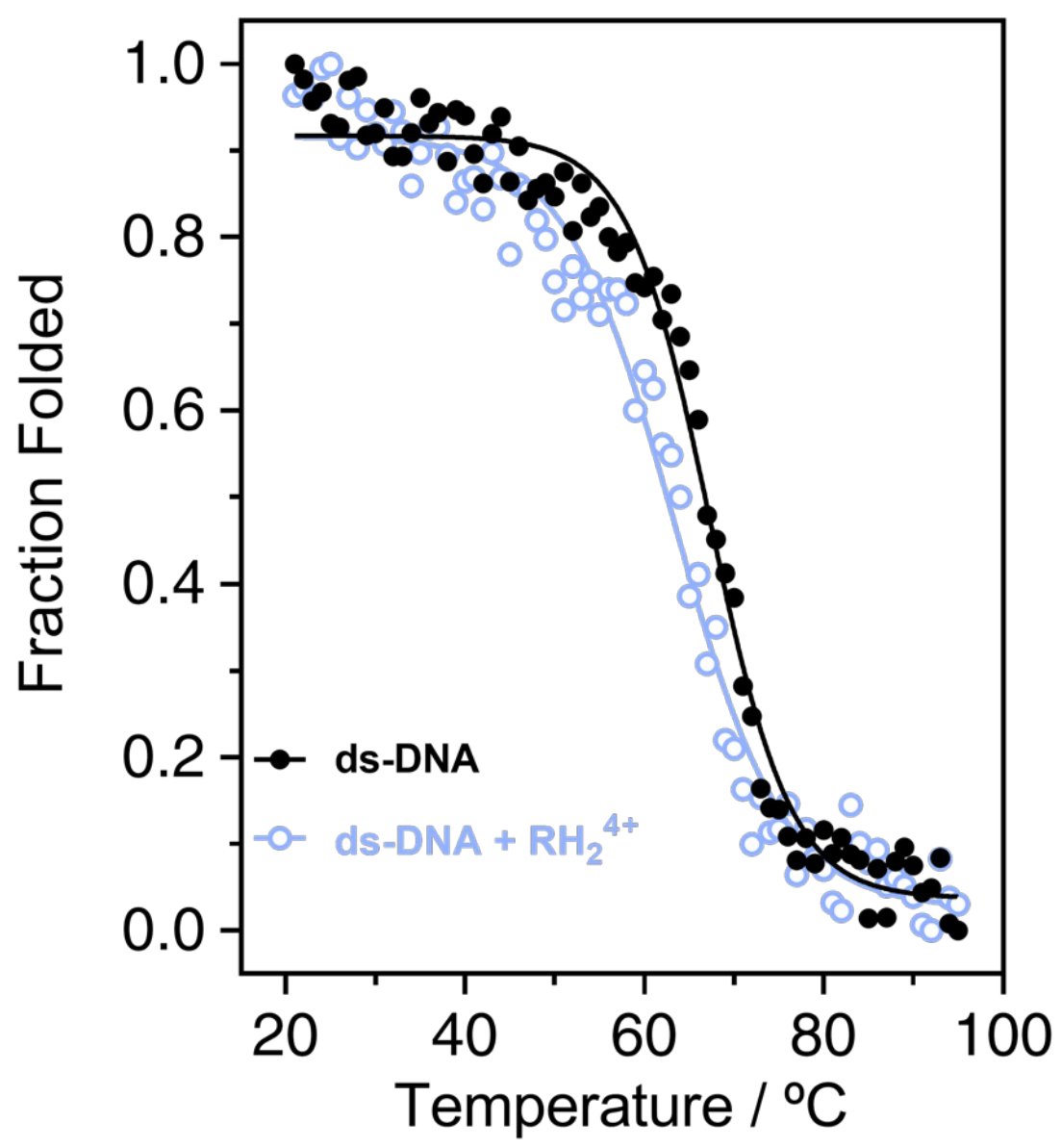

Figure S12. CD melting curves at 250 nm of a 10 µM ds-DNA solution in LCB4 in the absence (●) and in the presence of **RH_2^{4+}** (3 eq, ⊖).

*CD spectra of c-MYC and c-KIT2 in the presence of **RH_2^{4+}***

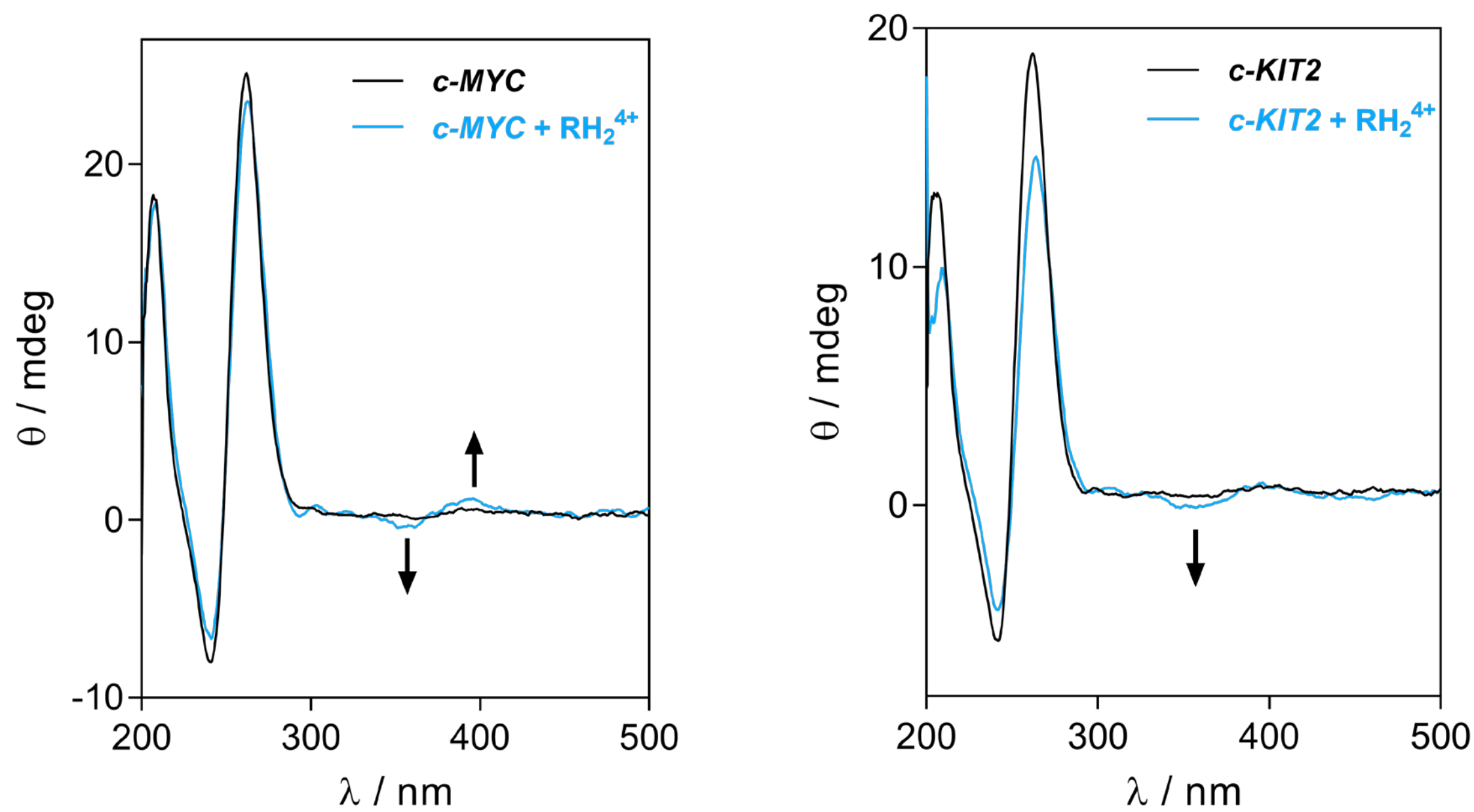

Figure S13. CD spectra of a 10 µM *c-MYC* (left) or *c-KIT2 (right)* solution in LB3 and LB2, respectively, and same solutions in the presence of 3 eq of **RH_2^{4+}**.

Red box interaction with FAM-c-MYC-TAMRA

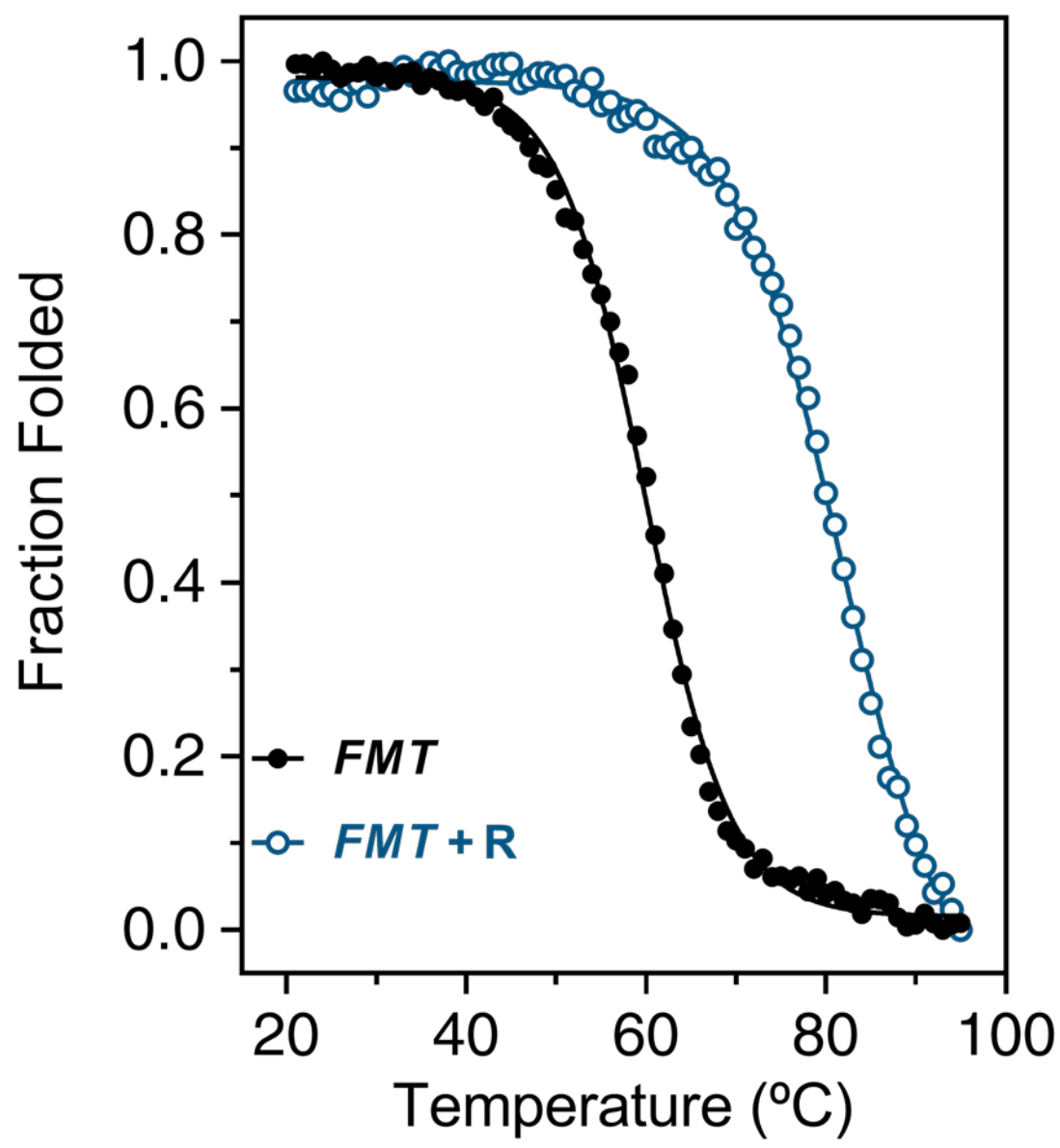

Figure S14. CD melting curves at 263 nm of a 10 μM FAM-*c-MYC*-TAMRA solution in LB6 (●), and a 10 μM FAM-*c-MYC*-TAMRA (*FMT*) solution with **R** (3 eq, ⊖) in LCB6.

FRET MELTING COMPETITION ASSAYS

FMT interaction with h-DNA

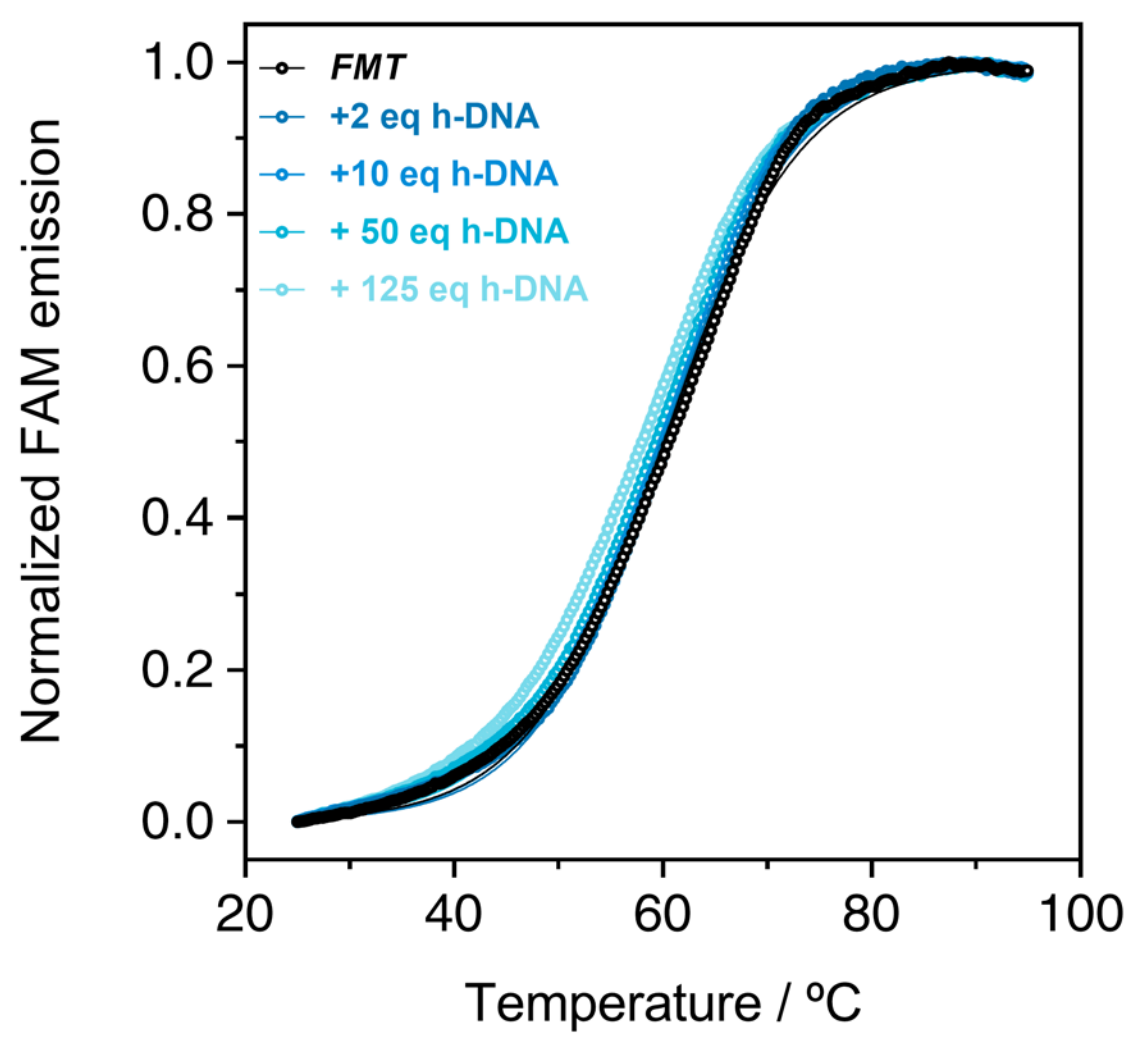

Figure S15. Normalized FRET-melting profiles of 0.2 μM FAM-*c-MYC*-TAMRA G4 (*FMT*) in LCB5 (⊖), and in the presence of 2 (⊖), 10 (⊖), 50 (⊖) or 125 eq (⊖) of h-DNA.

Competition of the FMT-R complex with ds-DNA

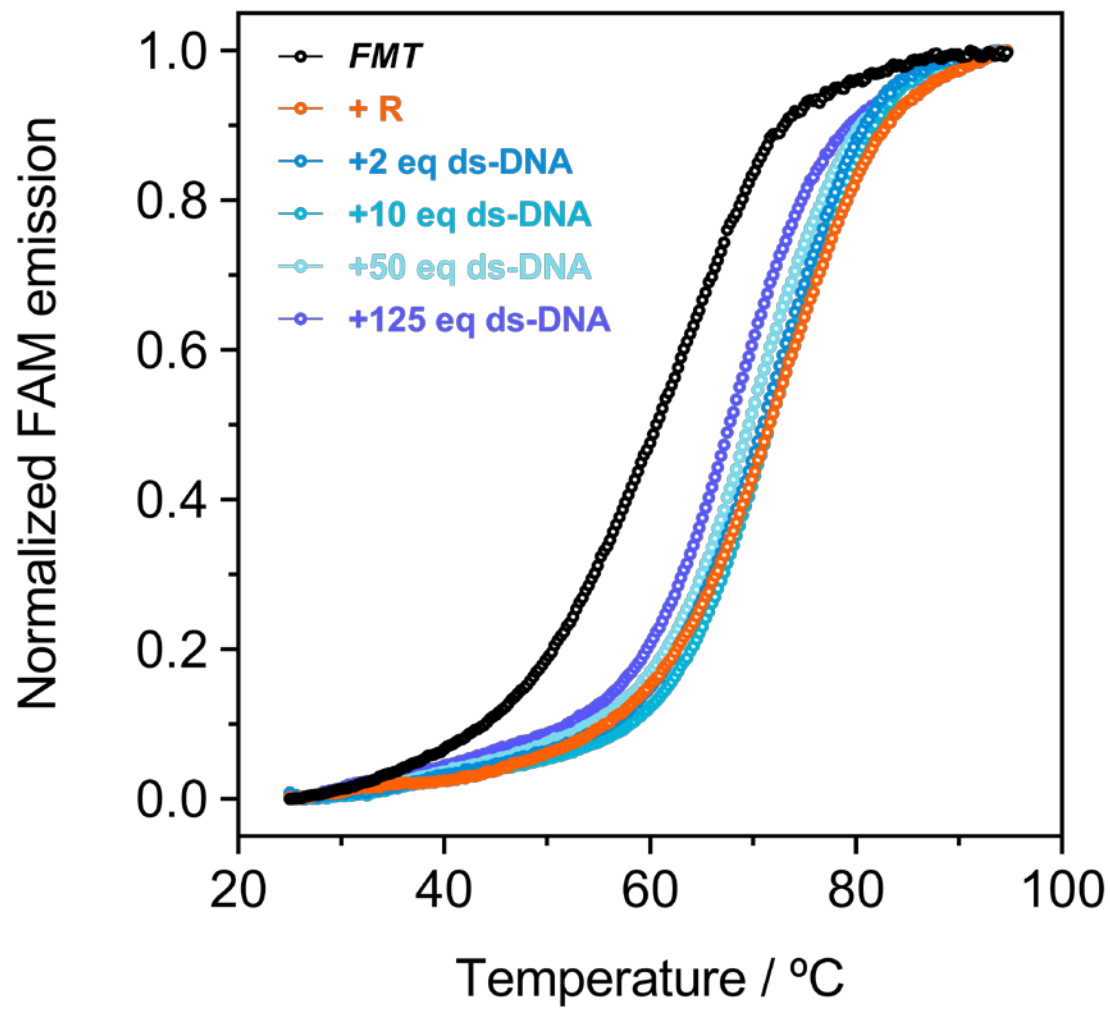

Figure S16. Normalized FRET-melting profiles of 0.2 µM FAM-*c-MYC*-TAMRA G4 (*FMT)* in 10 mM lithium cacodylate, 10 mM KCl, 90 mM LiCl pH 7.2 (LCB5) (⊖), in the presence of 3 eq of **R** (⊖), and after the addition of 2 (⊖), 10 (⊖), 50 (⊖) or 125 eq (⊖) of ds-DNA to the previous mixture.

ITC EXPERIMENTS

Red box interaction with c-MYC, c-KIT2 and h-DNA

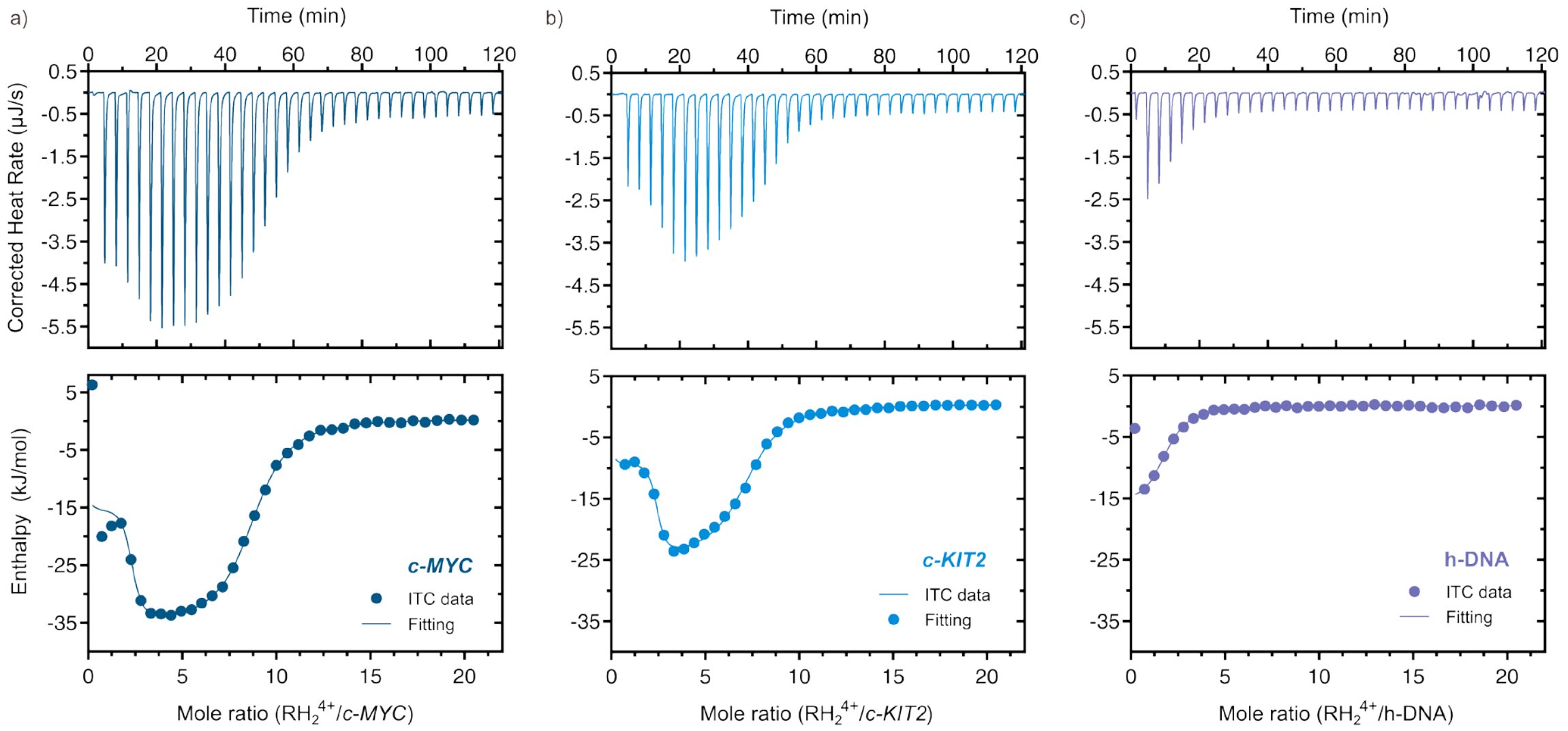

Figure S17.(a) ITC profile of a 10 μM *c-MYC* solution in LCB3, upon addition of successive aliquots of a 700 μM $\mathbf{RH_2}^{4+}$ solution in the same buffer. **(b)** ITC profile of a 10 μM *c-KIT2* solution in LCB2, upon addition of successive aliquots of a 700 μM $\mathbf{RH_2}^{4+}$ solution in the same buffer. **(c)** ITC profile of a 10 μM h-DNA solution in LCB2, upon addition of successive aliquots of a 700 μM $\mathbf{RH_2}^{4}$ solution in the same buffer.

Table S4. ITC data obtained for the $\mathbf{RH_2}^{4+}$ interaction with *c-MYC*, *c-KIT2*, and h-DNA at pH 6.3.

	c-MYC		*c-KIT2*		h-DNA
**Step**	1	2	1	2	1
n	2.3 ± 0.2	6.8 ± 0.6	2.1 ± 0.1	5.1 ± 0.1	2.2 ± 0.6
K_a[a]	(1.4 ± 0.6)×10^8	(6.1 ± 0.8)×10^5	(4.2 ± 5.0)×10^8	(4.3 ± 0.5)×10^5	(4.4 ± 0.8)×10^5
K_D[b]	(8.5 ± 4.2)×10^{-9}	(1.7 ± 0.2)×10^{-6}	(5.7 ± 4.1)×10^{-9}	(2.3 ± 0.3)×10^{-6}	(2.3 ± 0.4)×10^{-6}
ΔH[c]	−14.4 ± 1.6	−34.3± 2.2	−11.0 ± 1.4	−25.1 ± 0.5	−16.9 ± 1.4
$T\Delta S$[c]	31.8 ± 0.6	−1.3 ± 1.9	37.0 ± 2.0	7.1 ± 0.4	15.3 ± 1.8
ΔG[c]	−46.3 ± 1.2	−33.0 ± 0.1	−47.9 ± 3.0	−32.2 ± 0.3	−32.2 ± 0.5

[a] M^{-1}; [b] M; [c] kJ·mol^{-1}; SD calculated based on 3 replicates.

CELL ASSAYS

Cytotoxicity of Red box in SH-SY5Y, A375, HeLa, and HEK293T cell lines

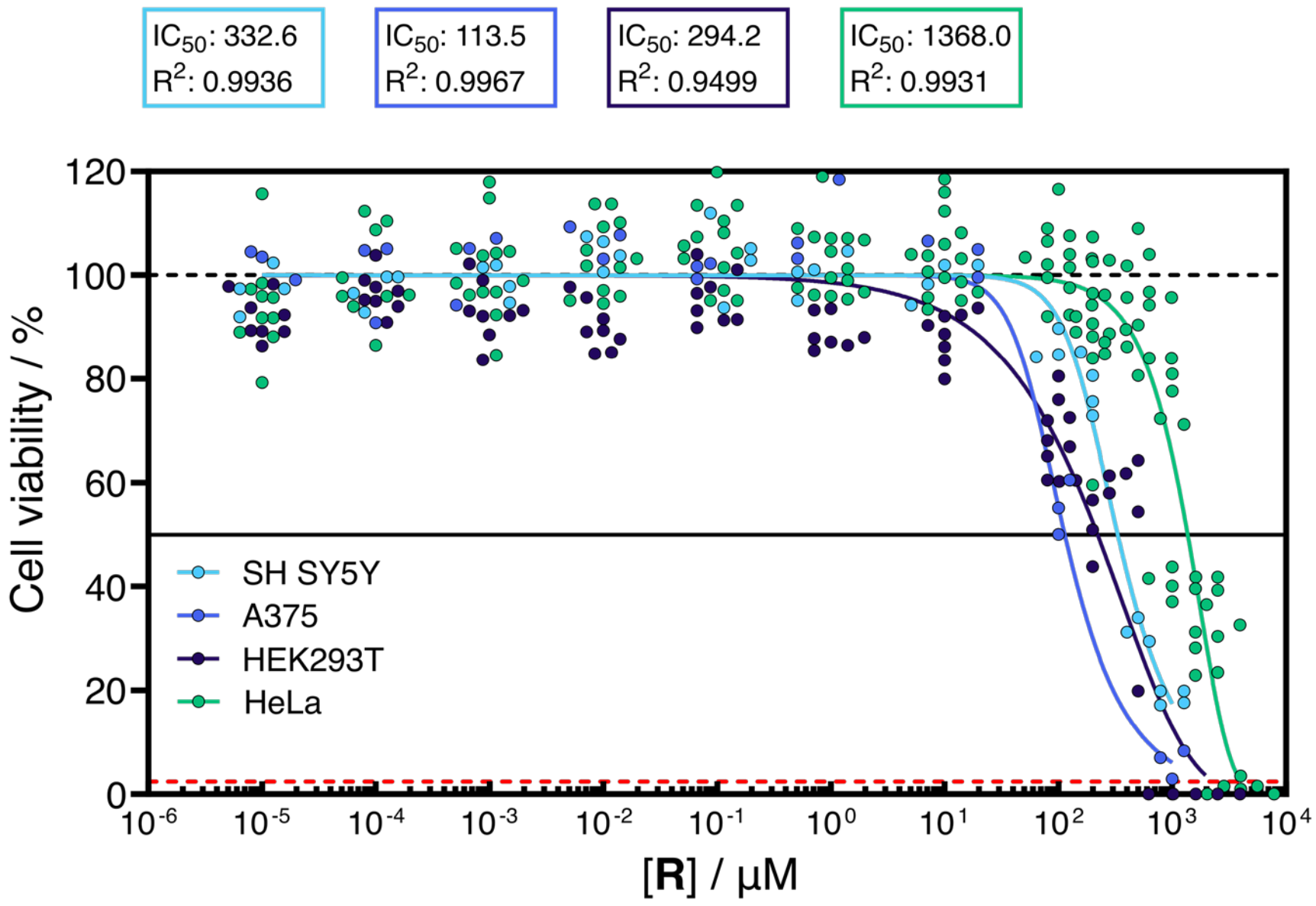

Figure S18. Cell viability of SH-SY5Y, A375, HeLa, and HEK293T cells after exposure to different **R** concentrations for 72h.

Basal expression levels of c-MYC, c-KIT, and hTERT in HEK293T cells

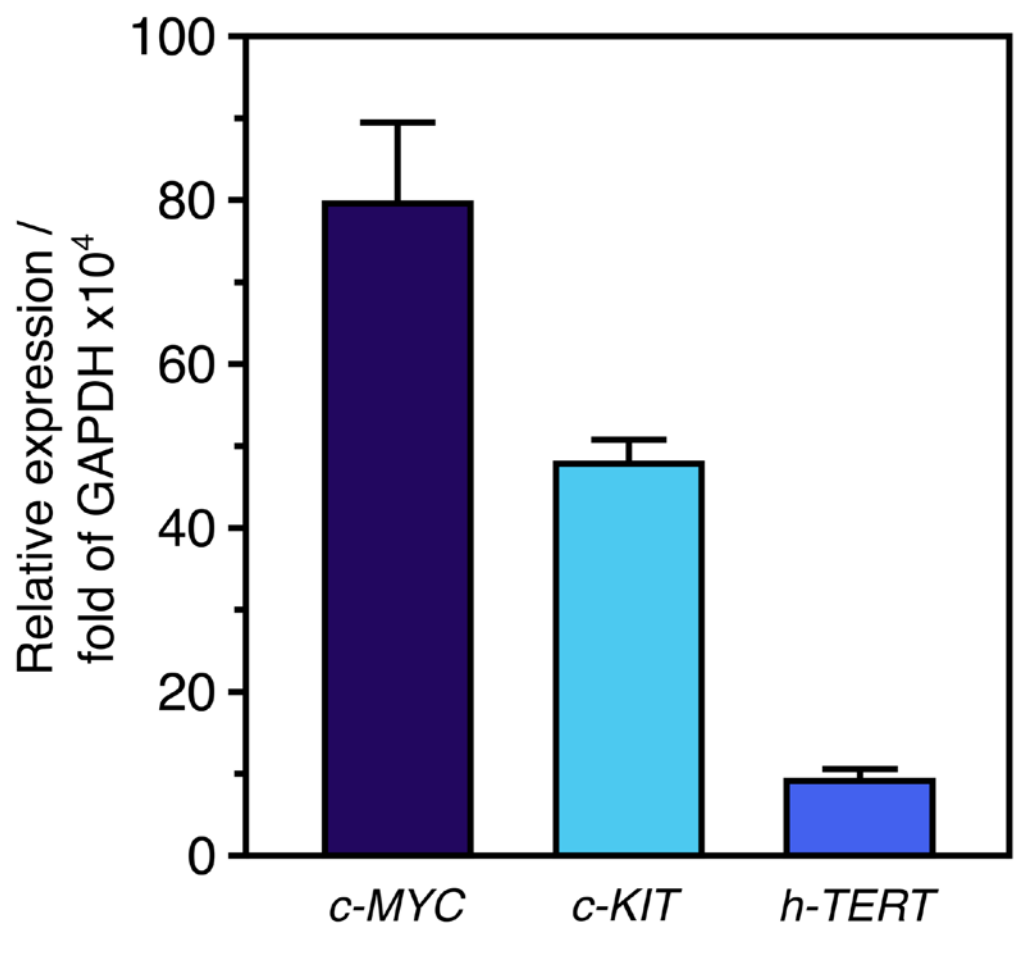

Figure S19. Non-normalized basal expression levels of *c-MYC*, *c-KIT*, and *hTERT* in untreated HEK293T cells. For optimal visual clarity and presentation of the data, the relative expression values (expressed as fold of *GAPDH*, ΔΔCt) were multiplied by 10,000 and are shown as $\times 10^4$ on the Y-axis).

REFERENCES

1 A. Blanco-Gómez, I. Neira, J. L. Barriada, M. Melle-Franco, C. Peinador and M. D. García, Thinking outside the "Blue Box": from molecular to supramolecular pH-responsiveness, *Chemical Science*, 2019, **10**, 10680–10686.

2 M. W. Pfaffl, A new mathematical model for relative quantification in real-time RT-PCR, *Nucleic Acids Res.*, 2001, **29**, e45.
